# An sRNA and Cold Shock Protein Homolog-Based Feedforward Loop Post-transcriptionally Controls Cell Cycle Master Regulator CtrA

**DOI:** 10.3389/fmicb.2018.00763

**Published:** 2018-04-24

**Authors:** Marta Robledo, Jan-Philip Schlüter, Lars O. Loehr, Uwe Linne, Stefan P. Albaum, José I. Jiménez-Zurdo, Anke Becker

**Affiliations:** ^1^LOEWE Center for Synthetic Microbiology and Faculty of Biology, Philipps-Universität Marburg, Marburg, Germany; ^2^Grupo de Ecología Genética de la Rizosfera, Estación Experimental del Zaidín (CSIC), Granada, Spain; ^3^LOEWE Center for Synthetic Microbiology and Faculty of Chemistry, Philipps-Universität Marburg, Marburg, Germany; ^4^Bioinformatics Resource Facility, Center for Biotechnology, Universität Bielefeld, Bielefeld, Germany

**Keywords:** non-coding RNAs, cell cycle, post-transcriptional control, RNA binding proteins, CtrA, *Sinorhizobium meliloti*

## Abstract

Adjustment of cell cycle progression is crucial for bacterial survival and adaptation under adverse conditions. However, the understanding of modulation of cell cycle control in response to environmental changes is rather incomplete. In α-proteobacteria, the broadly conserved cell cycle master regulator CtrA underlies multiple levels of control, including coupling of cell cycle and cell differentiation. CtrA levels are known to be tightly controlled through diverse transcriptional and post-translational mechanisms. Here, small RNA (sRNA)-mediated post-transcriptional regulation is uncovered as an additional level of CtrA fine-tuning. Computational predictions as well as transcriptome and proteome studies consistently suggested targeting of *ctrA* and the putative cold shock chaperone *cspA5* mRNAs by the *trans-*encoded sRNA (*trans-*sRNA) GspR (formerly SmelC775) in several *Sinorhizobium* species. GspR strongly accumulated in the stationary growth phase, especially in minimal medium (MM) cultures. Lack of the *gspR* locus confers a fitness disadvantage in competition with the wild type, while its overproduction hampers cell growth, suggesting that this riboregulator interferes with cell cycle progression. An eGFP-based reporter *in vivo* assay, involving wild-type and mutant sRNA and mRNA pairs, experimentally confirmed GspR-dependent post-transcriptional down-regulation of *ctrA* and *cspA5* expression, which most likely occurs through base-pairing to the respective mRNA. The energetically favored secondary structure of GspR is predicted to comprise three stem-loop domains, with stem-loop 1 and stem-loop 3 targeting *ctrA* and *cspA5* mRNA, respectively. Moreover, this work reports evidence for post-transcriptional control of *ctrA* by CspA5. Thus, this regulation and GspR-mediated post-transcriptional repression of *ctrA* and *cspA5* expression constitute a coherent feed-forward loop, which may enhance the negative effect of GspR on CtrA levels. This novel regulatory circuit involving the riboregulator GspR, CtrA, and a cold shock chaperone may contribute to fine-tuning of *ctrA* expression.

## Introduction

Bacteria, just like any other type of propagating cells, require robust mechanisms to faithfully replicate their genetic material and partition it to their progeny, even in fluctuating environments. To meet this challenge, bacteria have evolved diverse resilient regulatory mechanisms that guarantee a tight control of cell cycle progression while allowing for integration of environmental cues. In α-proteobacteria, cell cycle architecture shows conserved features, but also a high diversification (Brilli et al., 2010). The essential cell cycle regulator CtrA is broadly conserved in this group of bacteria and has a key role in governing cell cycle progression (Quon et al., 1996; Brilli et al., 2010). The role of CtrA has been best studied in *Caulobacter crescentus*. CtrA negatively controls initiation of DNA replication, but also transcriptionally regulates genes primarily involved in late cell cycle events, motility, and polar morphogenesis in this asymmetric bacterium, which divides in a stalked and a swarmer cell.

CtrA underlies multiple levels of transcriptional and post-translational regulation, the latter including phosphorylation and proteolysis (Domian et al., 1997; Reisenauer and Shapiro, 2002; Biondi et al., 2006; Iniesta et al., 2006; Heinrich et al., 2016). Transcriptional control of *ctrA* is based on a regulatory circuit mainly involving DnaA and GcrA (Holtzendorff et al., 2004; McAdams and Shapiro, 2009). DnaA initiates replication and activates a multitude of genes related to nucleotide biogenesis, polar morphogenesis and cell division, including *gcrA*. GcrA regulates genes related to DNA metabolism and segregation, and activates transcription of *ctrA*, thus providing a feedback mechanism. After translation, CtrA needs to be activated by phosphorylation, which is mainly controlled by the response regulator DivK. Altogether, these diverse mechanisms build an extensive circuitry that coordinates the cyclic occurrence and activity state of CtrA throughout the cell cycle.

While the core functionalities of this circuitry are understood in much molecular detail, considerably less is known about regulatory mechanisms tuning this cell cycle control to adapt to environmental conditions. Recent studies have unveiled the role of other regulators, such as the second messenger c-di-GMP and the alarmone (p)ppGpp in controlling CtrA levels in *C. crescentus* (Smith et al., 2014; Sanselicio et al., 2015). In *Sinorhizobium meliloti*, a plant symbiotic α-proteobacterium, the small non-coding RNA EcpR1 post-transcriptionally modulates expression of *dnaA* and *gcrA* under stress conditions (Robledo et al., 2015). This finding added an additional layer to the mechanisms that contribute to interlinking stress responses with the cell cycle machinery in bacteria. Untranslated small RNAs (sRNAs) are widespread post-transcriptional regulators that modulate fundamental processes of bacterial physiology in response to environmental conditions. Bacterial *tran*s-encoded sRNAs (*trans*-sRNAs) usually modulate mRNA translation and stability by pairing to the 5′-untranslated region (UTR), usually occluding the ribosome binding site (Waters and Storz, 2009). This interaction is often assisted by RNA binding proteins (Gottesman and Storz, 2011) and contributes to fine-tune the intracellular levels of proteins, thereby facilitating bacterial adaptation to changing niches.

Although our knowledge of sRNA functions in *S. meliloti* is still in its infancy, this non-coding transcriptome is one of the best known among α-proteobacteria in terms of structure, conservation, and functional characterization (Jiménez-Zurdo et al., 2013; Becker et al., 2014; Jiménez-Zurdo and Robledo, 2015). In this study, we took advantage of the comprehensive catalog of *trans*-sRNAs in this organism (Schlüter et al., 2010, 2013) and screened for sRNAs targeting the *ctrA* mRNA. Here, we report the *trans*-sRNA GspR to inhibit cell proliferation and demonstrate that it is able to post-transcriptionally modulate expression of the cell cycle master regulatory gene *ctrA*, and *cspA5*, which codes for a cold shock chaperone. We also show that CspA5 positively influences *ctrA* expression, thereby enabling a feed-forward loop composed of CtrA, CspA5, and the stress-induced *trans*-sRNA GspR, which may contribute to the regulation of CtrA levels.

## Materials and Methods

### Prediction of sRNA-mRNA Interactions

Computational predictions of sRNA-mRNA interactions, either using mRNA or sRNA as query sequences, was performed at a genome-wide scale applying IntaRNA and CopraRNA with standard parameters (Wright et al., 2014). The full-length GspR homologous sequences from *S. meliloti* 1021 (NC_003047, our reference genome), *S. meliloti* BL225C (NC_015590), *Sinorhizobium medicae* WSM419 (NC_009636), and *Sinorhizobium fredii* NGR234 (NC_012587) were used as query for CopraRNA. sRNA secondary structures were predicted with RNAfold (Gruber et al., 2008) and represented with VARNA (Darty et al., 2009).

### Bacterial Strains and Cultivation

Supplementary Table S1 lists all bacterial strains and plasmids used in this study. *E. coli* strains were grown at 37°C in LB medium and rhizobia either in complex tryptone yeast (TY) medium (Beringer, 1974), defined minimal medium (MM; Robertsen et al., 1981), or low phosphate (0.1 mM) MOPS-buffered MM (Zhan et al., 1991) at 30°C with agitation (200 rpm). Solid media were supplemented with antibiotics when required to the following final concentrations (mg/ml): streptomycin (Sm) 600; tetracycline (Tc) 10; gentamycin (Gm) 40; and kanamycin (Km) for *E. coli* and *Agrobacterium* 50 and for *Sinorhizobium* strains 180. The antibiotic concentration was reduced to 50% in liquid cultures. Unless other conditions are indicated, 1 mM IPTG was added to exponential phase cultures (OD_600_ of 0.4–0.45). Stress conditions were applied to exponentially growing cultures as previously described (Schlüter et al., 2013) and cells were harvested 1 h later. For growth assays, bacteria carrying the corresponding sRNA overproduction construct were grown to the indicated OD_600_ and 100 μl of IPTG-treated and untreated cultures were transferred to a 96 well microtiter plate to measure OD_600_ in a VICTOR Multilabel Plate Reader (PerkinElmer). Error bars indicate the standard deviation of at least 10 replicates growing in the same plate. Growth curves were repeated at least two times with similar results. Symbiotic assays with *Medicago sativa* plants were basically performed as described before (Robledo et al., 2015).

### Strain Construction

Primers used for cloning were designed based on the *S. meliloti* strain Rm1021 genome data (Galibert et al., 2001) and listed in Supplementary Table S2. Transcriptional fusions of promoterless *egfp* to the putative promoters of *gspR* and *SmelC776* (up to 188 and 229 nt upstream of the predicted TSS, respectively) were constructed by inserting the promoter regions as a SpeI-XbaI fragment in the replicative middle copy plasmid pBBegfp (Robledo et al., 2017). Translational fusion of *egfp* to predicted target genes were constructed by inserting PCR amplified fragments comprising the 5′ region from the native TSS (Schlüter et al., 2013) to the start codon and up to 33 further codons as XbaI (BglII)-NheI fragments into plasmid pR_EGFP.

Marker-free deletion of the *gspR-smelC776* locus by SOEing and construction of the IPTG inducible *sinR*-*sinI* based overexpression system of GspR and SmelC776 were basically performed as described before (Robledo et al., 2015). The deletion mutant lacks a 273 nucleotide stretch from 99 nt upstream the *gspR* TSS to 2 nt upstream the start of *SmelC776*. For induced overproduction of either SmelC776 or GspR, the encoding DNA regions were cloned into the middle-copy number plasmid pSRKKm under control of the SinR-controlled P*sinI* promoter in the *sinR sinI* Rm2011 mutant strain Sm2B2019, which was used to avoid interference with endogenous *sinR* and *sinI* alleles involved in quorum sensing. Induction of the P*sinI* promoter was controlled by IPTG-inducible expression of *sinR* included in the overexpression plasmid. Since sRNA genes were directly fused to the TSS of P*sinI*, sRNA transcription started with activation of P*sinI* (Becker et al., 2014; Robledo et al., 2015). In all sRNA overexpression assays, IPTG-driven transcription of the unrelated SmelC812 sRNA gene from plasmid pSKControl^+^ was used as negative control (Robledo et al., 2015). SmelC812 is encoded antisense to the 5′ UTR of a transposase gene and, to our knowledge, overproduction of this sRNA has no negative side effects (Schlüter et al., 2010). For *cspA5* complementation, the full-length gene was placed under control of the IPTG inducible P_lac_ promoter in plasmid pSRKGm.

### RNA Isolation and Northern Blot Hybridization

Total RNA including the sRNA fraction (50-250 nt) was isolated from bacterial cultures using the miRNeasy mini Kit (Qiagen). Initially, cell pellets were resuspended in 700 μl QIAzol Lysis Reagent (Qiagen) and then transferred to grinding tubes (soilGen). Cell destruction occurred via mechanical grinding using the X-Ribolyzer system (MP Biomedicals). After centrifugation, the supernatant was used for further treatment according to the manufacturer’s instructions (miRNeasy mini Kit, Qiagen). DNaseI digestion was applied according to the user manual instructions (Fermentas). RNA quality and concentration was measured using the NanoDrop2000 (Peqlab).

For northern blot, RNAs samples were separated on 6% polyacrylamide/7 M urea gels and transferred to nylon membranes by semi-dry electroblotting. 5′-end radiolabeled oligonucleotide probes (Supplementary Table S2) were used for hybridization as described (Robledo et al., 2018). To estimate RNA size, an RNA molecular weight marker (NEB) was included.

### Microscopy

Bacteria were examined using a Nikon Eclipse Ti-E by differential interference contrast and epifluorescence basically as described before (Frage et al., 2016).

### cDNA Synthesis and Microarray Hybridization

cDNA synthesis, microarray processing, sample hybridization, and image acquisition were performed as described previously applying the Sm14kOLI microarray that carries 50 –70 mer oligonucleotide probes directed against coding regions and both strands of the intergenic regions (IGR) (*Sinorhizobium meliloti* Rm1021 Sm14kOLI; Bahlawane et al., 2008). Probes in the IGR were separated by approximately 50–100 nt. The analysis of microarray images was performed with ImaGene 6.0 software (BioDiscoveries). Lowess normalization and significance test (*p*-value adjustment based on fdr) were performed with the EMMA software (Dondrup et al., 2009). The *M*-value represents the logarithmic ratio between both channels. The A-value represents the binary logarithm of the product of the intensities of both channels. Transcriptome data are available at ArrayExpress (Accession No. E-MTAB-3775).

### Proteome Sample Preparation

Bacteria were grown in MOPS medium with 21 mM of NH_4_Cl as nitrogen source instead of sodium glutamate. Notably, for all cultivations of GspR^+^ cells the ^15^N isotope of NH_4_Cl (Cambridge Isotope Laboratories) was used. Equal amounts of Control^+^ and GspR^+^ cells were pooled, harvested, and finally pelletized. Pellets were resuspended in 5–10 ml lysis buffer (10 mM MgCl_2_, 1 mM CaCl_2_, 50 μg/ml DNaseI, 50 μg/ml RNAseA, 20 mM Tris–HCl, pH = 8). Mechanical cell disruption was applied via three passages through a French press followed by centrifugation at 2,000 *g* for 2 min at 4°C. Ultracentrifugation of the supernatant was performed at 160,000 *g* for 1 h at 4°C to separate membrane (pellet) and cytoplasmic (supernatant) fractions. Pellets were resuspended in ∼5 ml lysis buffer. The resolved membrane and the cytoplasmic fraction were lyophilized. Dried protein samples (1 mg each) were resolved in 40–80 μl Tris loading buffer with 50 mM DTT, incubated at 70 °C for 10 min and treated with 120 mM Iodoacetamide 20 min in the dark at room temperature. Treated samples were separated in a SDS gel and each lane (200 μg protein) was sliced in ∼24 pieces (Sobrero et al., 2012). Gel-slices were dried in a SpeedVac (∼15 min/45°C). Trypsin treatment (0.1 μg trypsin per gel-slice) was applied overnight at 37 °C. After treatment, 200 μl MeCN was added to the sample for 30 min in an ultra-sonic bath. The supernatant was concentrated to dryness (Speedvac, 45°C) and finally dissolved in 25 μl 10% MeCN/0.1% TFA.

### Mass Spectrometry

Mass spectrometric analysis of the samples was performed using an Orbitrap Velos Pro mass spectrometer (ThermoScientific). An Ultimate nanoRSLC-HPLC system, equipped with a C18 nano RP column (particle size 1.8 μm) was connected online to the mass spectrometer through a Proxeon nanospray source. Depending on the concentration of the samples, 1–15 μl of the tryptic digest was injected onto a 2 cm × 300 μm PepMap C18 pre-concentration column. Automated trapping and desalting of the sample was performed at a flow rate of 6 μl/min using water/0.05% formic acid as solvent for 5 min. Separation of the tryptic peptides was achieved with the following gradient of water/0.045% formic acid (solvent A) and 80 % acetonitrile/0.05 % formic acid (solvent B) at a flow rate of 300 nl/min: holding 4 % B for 5 min, followed by a linear gradient to 45% B within 30 min and linear increase to 95% solvent B in an additional 5 min. The column was connected to a stainless steel nanoemitter and the eluent sprayed directly towards the heated capillary of the mass spectrometer using a potential of 2,300 V. A survey scan with a resolution of 60,000 within the Orbitrap mass analyzer was combined with at least 10 data-dependent MS/MS scans with dynamic exclusion for 30 s either using CID with the linear ion-trap or using HCD and orbitrap detection at a resolution of 7,500.

### Protein Identification and Quantification

Data sets were imported into the internet application QuPE (Albaum et al., 2009, 2011). A Mascot^TM^ (Perkins et al., 1999) search was conducted using a database that contained the complete proteome information of *S. meliloti* Rm1021 as well as an equally sized set of randomized amino acid sequences allowing for the later calculation of false discovery rates as suggested before (Reidegeld et al., 2008). Peptide tolerance was set to 10.0 ppm, MS/MS tolerance to 1,000.0 mmu, and two missed cleavage sites were allowed. Oxidation of methionine was allowed as a variable modification, and furthermore, a modification of arginine and lysine was introduced to account for a possible selected non-monoisotopic peak of a ^15^N-labeled precursor with a weight of approximately 1 Da (Zhang et al., 2009). Only hits having a score above Mascot’s own significance threshold (*P* < 0.05) were kept. In addition, false discovery rates were calculated in QuPE and required to be below *P* < 0.05. In total, 206,840 peptides were identified corresponding to 1,674 proteins. Proteome data were quantified using QuPE’s built-in algorithm using an ^15^N incorporation level of 98%. Rather strict parameters were employed (*r* > 0.4, isotopic distribution similarity >0.9) and results were filtered for a signal-to-noise value of at least 3.0. In total, 46,900 peptides were quantified accounting for 1,508 proteins. After quantification, all proteins were kept in the final result set which were represented either by at least two peptides with different amino acid sequences/charges or by peptides with the same amino acid composition but from two different samples. In summary, 512 proteins passed this filter criterion.

### Fluorescence Assays

Bacterial cells carrying the promoter test plasmids with transcriptional *gspR* promoter-*egfp* or SmelC776 promoter-*egfp* fusions were grown and measured as described before (Robledo et al., 2015, 2018). Reporter plasmids carrying translational fusions of the 5′ UTR and the first codons of target genes to *egfp* were transferred by conjugation to Sm2B2019DD harboring plasmids pSKControl^+^ or pSKGspR^+^. Three double transconjugants for each combination of inducible sRNA overproduction construct and target-*egfp* fusion were grown to exponential phase (OD_600_ of 0.5–0.6) and half volume of each culture were treated with 0.5 mM IPTG for 6 h. For estimation of the relative fitness, Rm2011 and 2011ecpR1 were labeled with mCherry or eGFP by single integration of either plasmid pKOSm or pKOSe (Robledo et al., 2015). Starter cultures were individually grown in MM overnight, diluted to OD_600_ of 0.005 and mixed at a 1:1 ratio in 5 ml of MM. Every 3 days, the eGFP and mCherry fluorescence of the cultures was measured and the mixed population was diluted 1,000-fold in fresh media. Treated and control cultures (100 μl) were transferred to a 96-well microtiter plate to measure OD_600,_ mCherry and/or eGFP-mediated fluorescence in the Infinite M200 Pro microplate reader (Tecan).

## Results

### sRNA GspR Is Predicted to Interact With the 5′ UTR of *ctrA* mRNA

**FIGURE 1:**
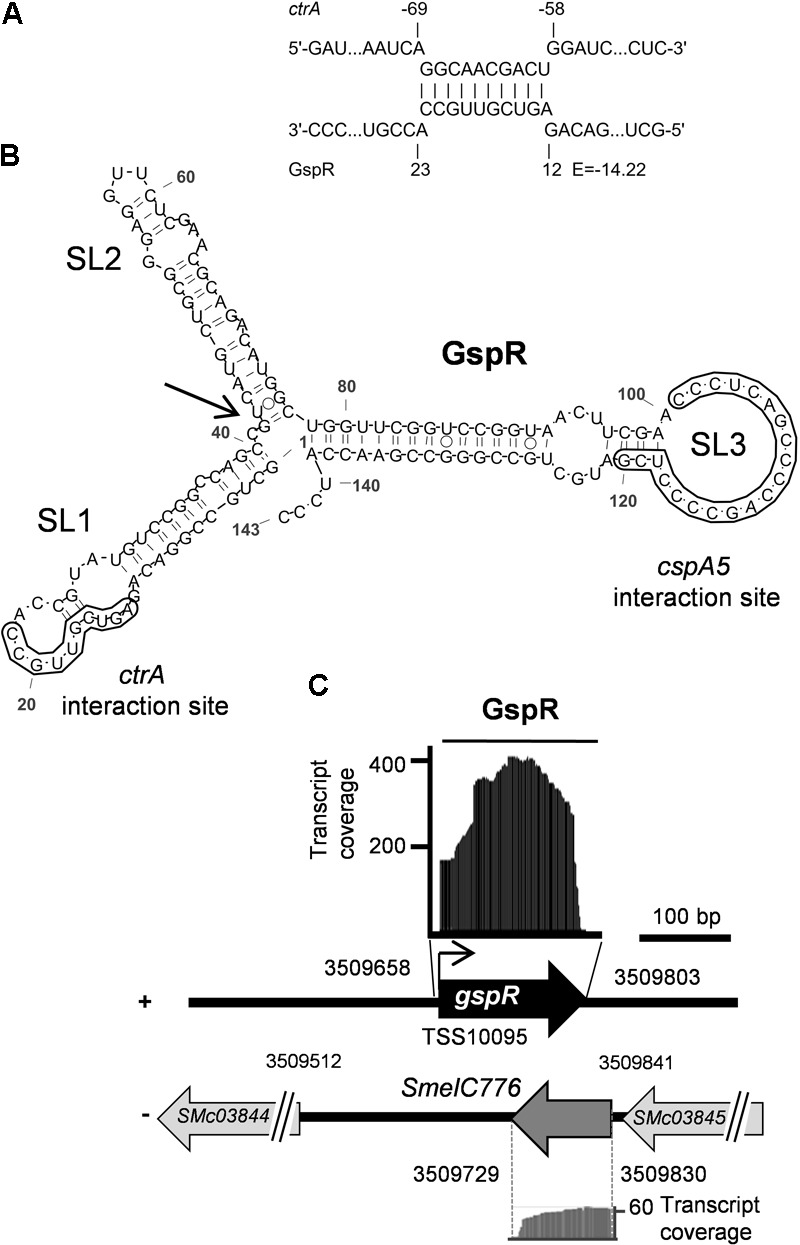
*ctrA* 5′ UTR is predicted to bind to GspR (SmelC775). **(A)** Predicted thermodynamically favored antisense interactions between GspR and *ctrA* mRNA. Numbers denote nucleotide positions relative to the TSS of *gspR* and the start codon of the putative target mRNAs. The predicted energy score (E) is indicated in kcal/mol. **(B)** Predicted secondary structure of the full-length GspR transcript with a minimum free energy of -66.30 kcal/mol exhibiting three independent stem loop (SL1 to SL3) structures. Nucleotide positions relative to the 5′-end are indicated. The SL1 and SL3 regions predicted to interact with the *ctrA* and the *cspA5* mRNAs, respectively, are boxed. **(C)** *gspR* genomic locus. Genes predicted on the complementary strand (-), including the SmelC776 locus are shown. Genome coordinates are indicated. RNAseq coverage profiles of both GspR and SmelC776 in *S. meliloti* Rm1021 are depicted. Black and gray areas represent coverage of GspR (SmelC775) and SmelC776, respectively, from samples enriched in processed transcripts (Schlüter et al., 2010). The angled arrow marks the GspR 5′-end (TSS10095) and the horizontal bar indicates the full-length 144 nt GspR sequence used for structure prediction.

In this study, we have taken a computational approach to identify sRNA candidates for post-transcriptional regulation of *ctrA* in *S. meliloti*. A stretch of the *ctrA* mRNA, encompassing the 5′ region from the most distal transcription start site (TSS) to the 50th codon (nucleotide positions -291 to +150 relative to the start codon), was used as query sequence to predict sRNAs as putative interaction partners. Hypothesizing that sRNAs controlling cell cycle-related target genes are phylogenetically conserved, we screened chromosomally encoded *trans*-sRNAs which have been previously found to be conserved in the order Rhizobiales (Reinkensmeier et al., 2011). This computational screen predicted the *S. meliloti trans*-sRNAs SmelC775 and SmelC291 (EcpR1) as potential regulators of *ctrA* (**Figures 1A,B**; Robledo et al., 2015). The putative interaction of SmelC291 (EcpR1) with the *ctrA* mRNA was previously investigated, although not experimentally confirmed (Robledo et al., 2015). Here, we focused on SmelC775.

SmelC775 was first identified by RNAseq in *S. meliloti* type strain Rm1021 as a 143-nt *trans-* sRNA (**Figures 1B,C**) and validated by northern blot (Schlüter et al., 2010). In this study, we renamed this sRNA GspR (Growth Stop Phenotype RNA) because of the phenotype caused by SmelC775 overproduction (see below). GspR is encoded approximately 145 kb distant from the chromosomal origin of replication in the intergenic region flanked by *SMc03844* and *SMc03845* (**Figure 1C**), both encoding conserved hypothetical proteins. RNAseq data identified the 100-nt transcript SmelC776 antisense to *gspR*. Both RNAs overlap by 74 nucleotides (Schlüter et al., 2010; **Figure 1C**). The predicted energetically most stable secondary structure of GspR consists of three stem-loop domains (SL1 to SL3, **Figure 1B**). The sRNA sequence predicted to base-pair with the *ctrA* mRNA comprises a continuous stretch of 10 nucleotides (E = -14.2 kcal/mol) mapping to GspR SL1 (nucleotide positions 13 to 22 of GspR) and to nucleotide positions -59 to -68 relative to the start codon of the *ctrA* mRNA (**Figure 1A**).

The GspR coding region was found to be conserved in various *Sinorhizobium*/*Ensifer* species including *S. meliloti, S. medicae, S. fredii, E. sojae, E. adherens*, and *S. americanum*. While SL2 shows slight sequence differences, SL1 and SL3 motifs are identical in all GspR homologs (Reinkensmeier et al., 2011), suggesting that these sequences may be involved in conserved interactions with target mRNAs. Therefore, the full-length sequences of GspR homologs from different *Sinorhizobium* species were also scanned for putative interactions with mRNAs as described in section “Materials and Methods.” This screen returned the *ctrA* mRNA as conserved GspR target. Identical GspR-*ctrA* mRNA interaction sites (**Figure 1A**) were predicted in the different *Sinorhizobium* species. This suggested functional conservation, which motivated us to further investigate this sRNA.

### GspR Accumulated in the Stationary Phase of Bacterial Growth

**FIGURE 2:**
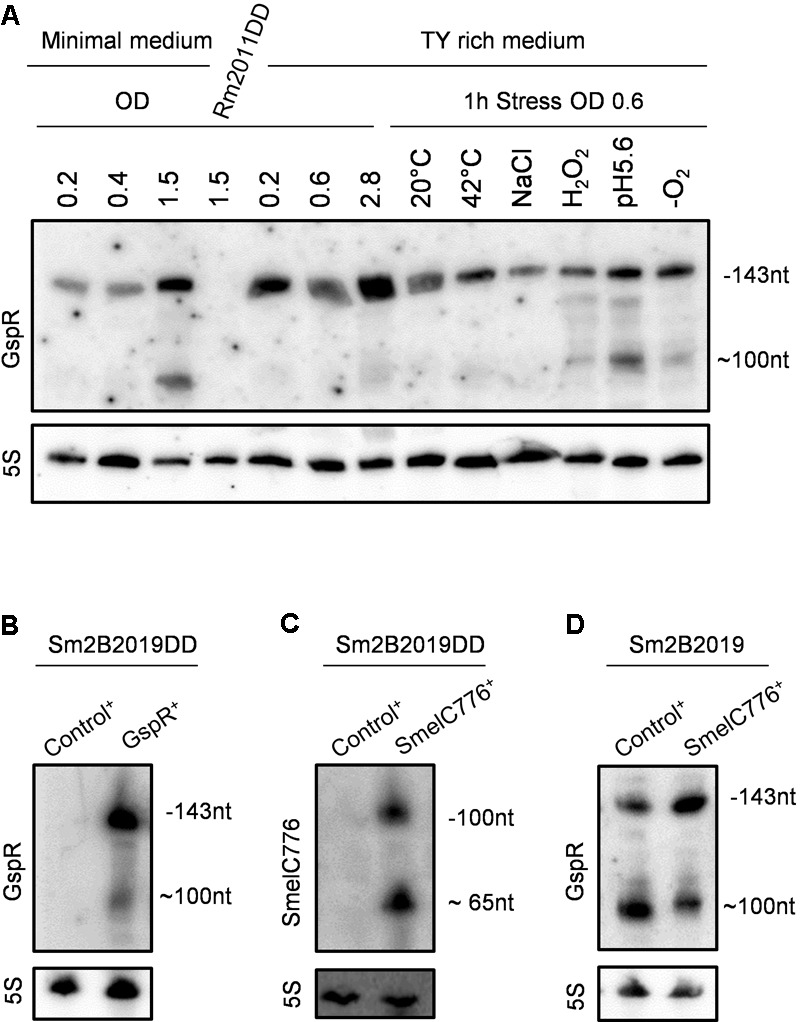
Accumulation of GspR is independent of SmelC776 expression. **(A)** Expression profiling of GspR. Northern blot detection of GspR abundance in the *S. meliloti* Rm2011 wild type under different growth and stress conditions in minimal and TY-rich media in exponential and stationary growth phase and in TY-rich medium at 20°C, cold stress; 42°C, heat stress; NaCl, 0.4 mM sodium chloride (osmotic stress); H_2_O_2_, 10 mM hydrogen peroxide (oxidative stress); pH 5.6, 20 mM MES (acidic stress); -O_2,_ microoxic conditions. Exposure times were optimized for each panel. 5S rRNA probing was used as RNA loading control. **(B–D)** Northern blot detection of GspR and SmelC776 RNA variants in Sm2B2019DD or Sm2B2019 strains carrying either pSKControl^+^ (Control^+^), pSKSmelC776^+^ (SmelC776^+^), or pSKGspR^+^ (GspR^+^), 4 h after induction with IPTG.

Previous northern blot hybridizations, detecting GspR with a double-stranded DNA probe, showed *gspR* expression in all the conditions tested (i.e., exponential growth phase in TY and GMX media, and cold, heat, and salt stresses) with just small variations in expression levels in the *S. meliloti* 2011 wild type (Schlüter et al., 2010). Since sRNA SmelC776, transcribed antisense to *gspR* (**Figure 1C**), may interfere with GspR hybridization, we used strand-specific antisense oligonucleotides to detect each sRNA independently in *S. meliloti* Rm2011. This wild-type strain is a close relative of the type strain *S. meliloti* Rm1021. SmelC776 hybridization signals were not clearly detected in any of the conditions tested (Supplementary Figure S1). However, northern hybridizations identified a dominant ∼143-nt GspR transcript and a shorter variant of ∼100 nucleotides, together with less abundant shorter GspR-derived RNA species under several growth conditions (**Figure 2A**). In light of previous RNAseq data (**Figure 1C**; Schlüter et al., 2010), the ∼100-nt processed form may constitute a GspR variant lacking SL1.

GspR-derived RNA species were detected in exponentially growing *S. meliloti* Rm2011 and accumulated during the stationary phase of growth, especially in MM cultures. However, the amount of GspR did not increase significantly in different stress conditions, i.e., cold, heat, salt, oxidative, acidic, and microoxic stresses, compared with the exponential growth condition in TY-rich medium (**Figure 2A**).

Previous analysis of dRNAseq data identified a primary 5′-end of GspR (TSS_10095, **Figure 1C**) but not of SmelC776 (Schlüter et al., 2013). The putative promoter regions of *gspR* and *SmelC776*, comprising nucleotide positions -159 to +29 and -221 to +8, respectively, relative to the corresponding TSS were fused to *egfp*. When cultured in TY or minimal media, Rm2011 carrying the SmelC776 upstream region-reporter fusion did not exceed background fluorescence derived from the control plasmid lacking a promoter upstream of *egfp.* These results suggest that the fused region has no promoter activity under the conditions tested and that SmelC776 is probably not a primary transcript. In agreement with the failure to detect a primary 5′ end by dRNAseq (Schlüter et al., 2010), this sRNA may rather be generated by post-transcriptional processing from the 3′ UTR of the upstream *SMc03845* mRNA.

When grown in TY rich or minimal media, wild-type strain Rm2011 carrying the P*_gspR_*-*egfp* promoter reporter fusion showed activities in exponentially growing bacteria (OD_600_ of 0.4 or 0.6). These activities slightly increased in the early stationary growth phase, more visible in minimal than in rich medium, matching the trend towards higher levels of GspR in the stationary growth phase, as revealed by northern hybridizations (Supplementary Figure S2A). The GspR 5′-end was not preceded by known *S. meliloti* promoter signatures. However, the *gspR* upstream region is 100% conserved in *S. meliloti* and *S. medicae* and 84% in *S. fredii* NGR234 strain (Supplementary Figure S2B).

### GspR Expression and Processing Are Likely Independent of SmelC776

The *gspR* and *SmelC776* coding regions are inversely oriented and overlap in their 3′ regions by 74 nucleotides (**Figure 1C**), suggesting mutual interference between the GspR and SmelC776 sRNAs. This hypothesis was tested by studying the effect of increased levels of one of these sRNAs on the other. To this end, the abundance of either of these sRNAs was increased by plasmid-based IPTG-induced overproduction, either in Sm2B2019 with the wild type *gspR*/*SmelC776* chromosomal locus, or in the *gspR*/*SmelC776* markerless deletion mutant Sm2B2019DD. This mutant, lacking the putative *gspR* promoter and the coding regions of both full-length sRNAs, was used as genetic background to avoid interference with the chromosomal locus. The SmB2019 background (Δ*sinRI*) was required to exclude interference with the plasmid-based inducible overexpression system (see section “Materials and Methods”). The control sRNA gene *SmelC812* similarly cloned in plasmid pSKControl^+^ was used as control in overexpression assays (Robledo et al., 2015).

Accordingly, *gspR* was not detected by northern hybridization of RNA from stationary phase cultures of Rm2011DD and Sm2B2019DD in MM (**Figures 2A,B**). Northern hybridizations detected two signals corresponding to 100-nt and ∼65-nt SmelC776 species upon IPTG-induced ectopic overexpression of *SmelC776* in Sm2B2019DD (**Figure 2C**). This processing pattern is unlikely to be driven by a possible antisense interaction with GspR, since it occurred independently of the presence of the chromosomal *gspR/SmelC776* locus (**Figure 2C**). The 100-nt processed form of GspR was also detected regardless the presence of the chromosomal locus (**Figure 2B**), further suggesting that mechanisms of GspR and SmelC776 biogenesis are unrelated. Hybridization with two pairs of riboprobes, each targeting a region within the overlapping stretch of GspR and SmelC776, and another specific to one of these transcripts, rendered the same pattern (data not shown). Finally, the overproduction of SmelC776 in Sm2B2019 cells did not alter processing or stability of GspR (**Figure 2D**). Taken all together, the biogenesis of these two sRNAs is probably mutually independent.

### The *gspR* Locus Confers a Fitness Advantage in *S. meliloti*

To study the biological function of GspR, motility, growth, morphology, and symbiotic phenotypes were monitored in *S. meliloti* strains lacking *gspR*. Both the *gspR/*SmelC776 deletion mutant Rm2011DD and the *gspR/*SmelC776*/ecpR1* triple mutant (i.e., containing an additional deletion in the locus coding for the cell-cycle related EcpR1 sRNA; Robledo et al., 2015) were not impaired in motility and they exhibited wild type-like growth in TY rich, MOPS (both defined or nutrient-limited) or MM. Cell viability (CFU/ml) after growth in MM until late stationary phase was also unaffected with respect to the wild type. Furthermore, the markerless *gspR/*SmelC776 deletion mutant was proficient in symbiosis with its host plant *Medicago sativa*, indistinguishable from the symbiotic phenotypes of the parental strain. Transposon insertions in *SMc03844* or *SMc03845* flanking the *gspR/*SmelC776 locus also showed wild type-like growth (data not shown).

In contrast, deletion of *gspR/*SmelC776 attenuates competitiveness as determined by a fitness growth assay against the Rm2011 wild type. For this experiment, strains were chromosomally tagged to constitutively express *egpf* or *mcherry* as previously done (Robledo et al., 2015). 2011 egfp cells were mixed in a ratio of 1:1 with either 2011 mCherry or 2011DD mCherry cultures in MM and grown until the late stationary phase, where the highest levels of GspR expression were detected (**Figure 2A**). Every 3 days of incubation, the eGFP:mCherry fluorescence ratio of the stationary cultures was measured (Supplementary Figure S3) and the mixed population was diluted 1,000-fold in fresh MM. After 3 days of cultivation, the ratios of fluorescent signals representing the proportions of the mixed strains were similar in all cultures. The wild-type control culture further maintained a similar ratio even after three consecutive sub-cultivations. However, the proportion of the 2011 egfp strain progressively increased over the 2011DD mCherry mutant, showing a 52% increment of the fluorescence ratio after the third sub-cultivation. These results indicate a lower fitness of the mutant in competition with the wild type.

### GspR Overproduction Hampers Cell Growth in *Sinorhizobium*

**FIGURE 3:**
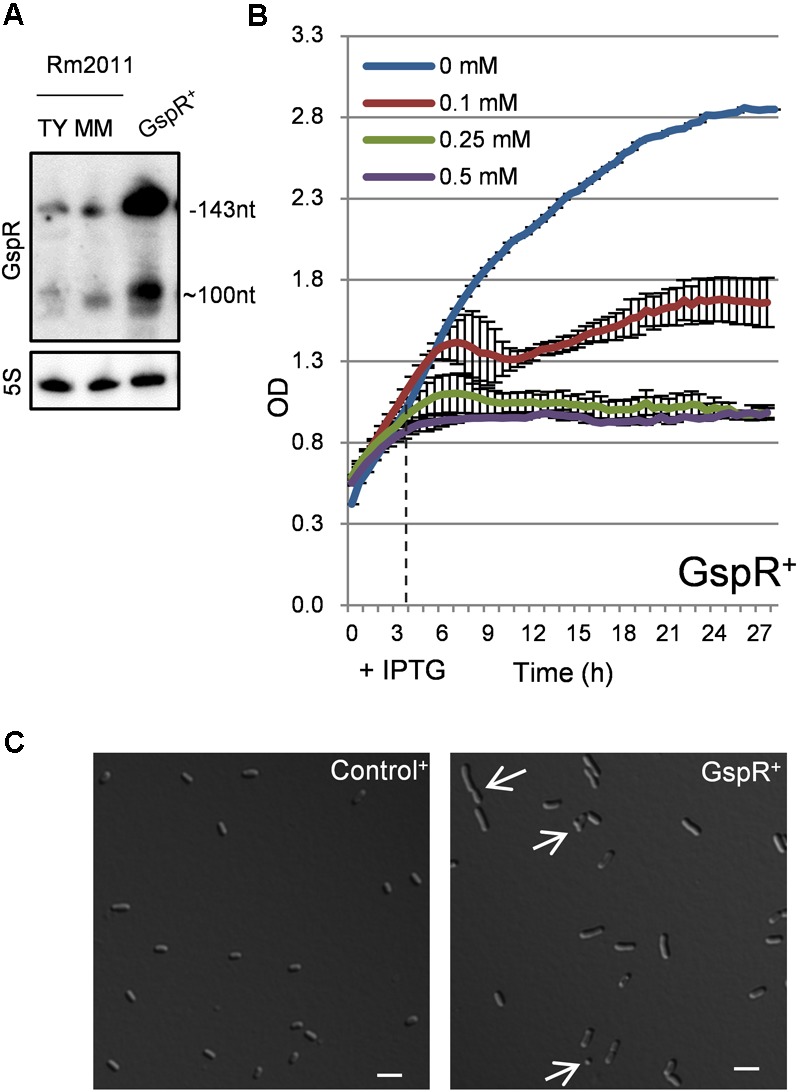
Growth stop phenotype associated to *gspR* overexpression. **(A)** Northern blot detection of GspR RNA variants in the *S. meliloti* Rm2011 wild type strain at the stationary growth phase in rich TY or minimal media (left) or in the Sm2B2019DD strain carrying pSKGspR^+^ (GspR^+^) 4 h upon induction with 0.5 mM IPTG. 5S hybridization signals are shown as loading controls. **(B)** Growth kinetics after addition of different IPTG concentrations to OD_600_ 0.4 TY-rich medium cultures of *S. meliloti* Sm2B2019DD cells carrying pSKGspR^+^ (0 h). Horizontal dashed line shows the start point of significant growth differences following addition of 0.5 mM IPTG (4 h). **(C)** Morphology of *S. meliloti* Sm2B2019DD strains overproducing GspR or the SmelC812 control antisense RNA 30 h post-induction with 0.5 mM IPTG. Arrows mark cells with abnormal morphology in GspR^+^ cultures (see text for details). Bars correspond to 2 μm.

To obtain further clues to the cellular role of the GspR sRNA, the GspR-overproducing *S. meliloti* strain was phenotypically characterized. IPTG-induced overexpression of plasmid-borne *gspR* in strain Sm2B2019DD, confirmed by northern hybridization, negatively affected growth (**Figures 3A,B**). While Sm2B2019DD carrying the *gspR* overexpression plasmid pSKGspR^+^ showed normal growth in absence of the inducer, growth was increasingly hampered in the presence of rising concentrations of the inducer and completely inhibited at 0.5 mM IPTG (**Figure 3B**). The delay in growth started approximately 4 h after induction, which corresponds to two *S. meliloti* cell cycles. Growth differences between induced and uninduced cultures were more pronounced when IPTG was added at lower cell densities and were less dramatic when induction was started at higher initial cell densities (Supplementary Figures S4A,B). Overexpression of other *S. meliloti* sRNAs (e.g., SmelC776, SmelCR01029, SmelCR01763, SmelB008 and SmelC045) using the same strategy did not affect growth (data not shown).

24 h post-induction with 0.5 mM IPTG, control cells showed the typical morphology of stationary phase cells, 1 to 2 μm in length, while GspR overproducing cells were heterogeneous in morphology (**Figure 3C**); 12.7% of the *gspR* overexpressing cells were abnormally long (>2 μm), 6.0% showed a round morphology (length <1 μm) and an additional 1.0% showed a branched morphology (**Figure 3C**, white arrows; sampling of 1,000 cells). In contrast, less than 0.5% of the control population showed abnormal cell morphologies. Time-lapse microscopy revealed that 50% of the cells inhibited in growth by *gspR* overexpression (induced by 0.5 mM IPTG) proceeded to cell division and resumed growth when transferred to fresh medium lacking the inductor. In comparison, 96% of equally treated Control^+^ cells resumed growth. Overproduction of *S. meliloti* GspR also led to cell growth arrest in *S. fredii* and *S. medicae*, both encoding GspR homologs, but not in *Agrobacterium tumefaciens*, which lacks a GspR homolog (Supplementary Figure S4C).

GspR sRNA has been reported to be Hfq-independent (Torres-Quesada et al., 2014). Growth arrest upon induced GspR overexpression was independent of the RNA chaperone Hfq and the C-terminal domain of the ribonuclease RNase E, i.e., *gspR* blocked growth in both the *hfq* deletion mutant Rm2011*hfq* and Rm2011*rne675* encoding an RNase E variant lacking the C-terminal domain (Baumgardt et al., 2014; Supplementary Figures S4D,E).

### GspR Regulates *cspA5* and *ctrA* mRNAs Through Distinct Stem-loops

Bacterial sRNAs typically target multiple mRNAs. To identify other putative GspR target genes, transcriptome and proteome profiling were performed 30, 60, and 90 min post-induction of GspR overproduction (Supplementary Figure S5, and Supplementary Data Sheet S1). The microarray experiments distinguished 56 (30 upregulated/26 downregulated) differentially expressed protein coding genes in the GspR^+^ strain in comparison to the control strain, together with eleven 5′-UTRs, two mRNA fragments, and several sRNAs. GspR-dependent changes in protein synthesis were observed for 19 (8/11) candidates. Together, transcriptome and proteome analysis identified 68 (34 up, 34 down) protein coding genes showing altered gene expression profiles in the pSKGspR^+^ strain (*M* value: ≥1, ≤-1). Global functional predictions showed that the majority of identified genes (∼34%, 23) are related to cellular metabolism of *S. meliloti* with a clear dominance in transport/metabolism of amino acids (7) and inorganic ions (8).

The following putative target genes were consistently identified by both computational predictions (CopraRNA prediction in *Sinorhizobium* with *P* < 0.05) and expression profiling experiments (significant decrease (*M* ≤ -1) in transcript or protein abundance, at least at one of the tested time points following *gspR* overexpression (Supplementary Data Sheet S1): *ctrA* [CopraRNA prediction *ctrA* 5′-UTR: *P* < 0.042; transcriptome profiling *ctrA*: *M* = -0.41 (60 min) and -0.50 (90 min); proteome profiling: *M* = -0.56 (30 min) and -1.14 (60 min)], *cspA5* [CopraRNA prediction *cspA5* 5′-UTR: *P* < 0.00024; transcriptome profiling *cspA5* 5′-UTR: *M* = -0.65 (30 min), -1.36 (60 min), and -0.67 (90 min)], and *SMc02819* [CopraRNA prediction *SMc02819* : *P* < 0.00014; proteome profiling *SMc02819*: *M* = -1.50 (30 min)].

GspR-mediated translational control of these candidate target mRNAs was tested by a two-plasmid assay (Torres-Quesada et al., 2013; Robledo et al., 2015). To this end, the 5′-UTR of the putative target gene (Schlüter et al., 2013), containing the predicted GspR interaction sequence, and a short stretch of the corresponding coding region, was translationally fused to *egfp* under the control of the constitutive synthetic P_Syn_ promoter (Giacomini et al., 1994) to generate reporter constructs in plasmid pR_EGFP. A *gcrA* reporter construct (Robledo et al., 2015), not regulated by GspR, was used as negative control (Supplementary Figure S6). These reporter plasmids were mobilized to Sm2B2019DD GspR^+^ and *S. meliloti* Sm2B2019DD Control^+^, carrying plasmid-borne inducible *gspR* and *SmelC812* (control sRNA gene; Robledo et al., 2015) expression constructs, respectively. Post-transcriptional effects were assessed by determining fluorescence of the reporter fusion protein upon induced sRNA expression. All assays were performed under conditions ensuring comparable growth of the reporter strains (see section “Materials and Methods”).

#### SMc02819

Its protein product is homologous to endoribonuclease T2. A 15-nt stretch overlapping the coding sequence (positions +36 to +51) was predicted to interact with GspR SL3 (E = -18.09 kcal/mol). We were unable to test *SMc02819* for GspR-mediated regulation because the reporter construct p*SMc02819*_-136+99_-*egfp* displayed hardly detectable fluorescence.

#### cspA5

**FIGURE 4:**
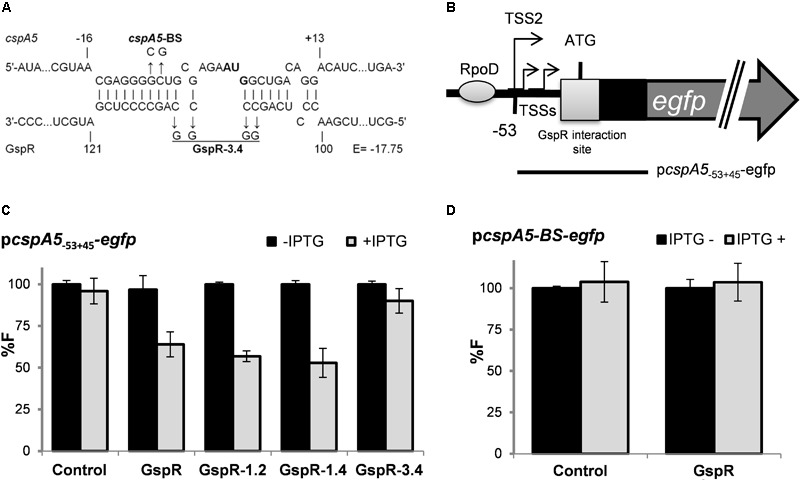
GspR SL3 represses *cspA5* post-transcriptionally. **(A)** Predicted duplexes between GspR and *cspA5* mRNA. Positions are denoted relative to the AUG; A is +1. The predicted energy score (E) is indicated in kcal/mol. The nucleotide exchanges in the predicted interaction regions of *cspA5* (*cspA5*-BS) and GspR (GspR 3.2 and GspR 3.4) mRNAs are indicated in bold. **(B)** Schematic representations of the genomic regions and the fragment (indicated by bars) translationally fused to *egfp*. The potential *cspA* RpoD-dependent promoter is indicated by a gray circle and GspR-interaction site by a box. **(C,D)** Means of relative fluorescence intensity values of Sm2B2019DD co-transformed with overexpression plasmids carrying control *SmelC812, gspR*, or its mutant variants; and P*cspA5_-53+45_-*egfp C) or P*cspA5*-BS-egfp. **(D)** Reporter plasmids carrying either the native 5′UTR encoding sequences derived from *cspA5* or a variant with 2 mutations in the predicted GspR binding sites (BS). The standard deviation represents at least three independent determinations of three double transconjugants grown in three independent cultures. Specific activities were normalized to the levels of the strain carrying the vector with the control RNA gene without IPTG added to yield percent relative fluorescence (% F).

Homologs of *cspA5* encode cold shock proteins, which are generally, but not exclusively, induced upon a temperature downshift affecting mRNA structure stability and RNase recognition to counteract the physiological effects of temperature changes (Barria et al., 2013). Discontinuous base-pairing of GspR SL3 with a 27-nt stretch overlapping the Shine-Dalgarno and the start codon sequences of the *cspA5* mRNA (nt position -16 to +13 relative to the AUG) was predicted (*E* = -17.75 kcal/mol; **Figures 4A,B**). Four different TSSs have been assigned to the *cspA5* mRNA (Schlüter et al., 2013). To design a reporter construct, we selected the second TSS, which is located 53 nt upstream of the AUG and preceded by a RpoD promoter signature (Schlüter et al., 2013). The reporter construct comprised the *cspA5* 5′-UTR starting with TSS2 and the first 48 nt of the coding region fused to *egfp* (plasmid p*cspA5*_-53+45_-egfp; **Figure 4B**). This construct was used to assess the regulatory effect of GspR and a series of mutant variants on *cspA5* by the double-plasmid assay. Induced overexpression of *gspR* reduced p*cspA5*_-53+45_-egfp mediated fluorescence by 36% compared to the control. Overproduction of GspR mutant variants carrying changes of 2 or 4 nt in SL1 similarly repressed activity of this *cspA5* reporter construct, while GspR-3.4, carrying mutations in SL3, did not influence reporter activity (**Figure 4C**). This further supports that the GspR*-cspA5* mRNA interaction region resides in SL3 of the sRNA. Accordingly, changes of 2 nt in the predicted target region within the *cspA5* 5′-UTR of the reporter construct (p*cspA5*-BS-egfp) abolished the negative effect caused by *gspR* overexpression (**Figure 4D**). Therefore, GspR SL3 seems to canonically regulate *cspA5* by antisense pairing with a nucleotide stretch located closely upstream of the start codon in the mRNA.

#### ctrA

**FIGURE 5:**
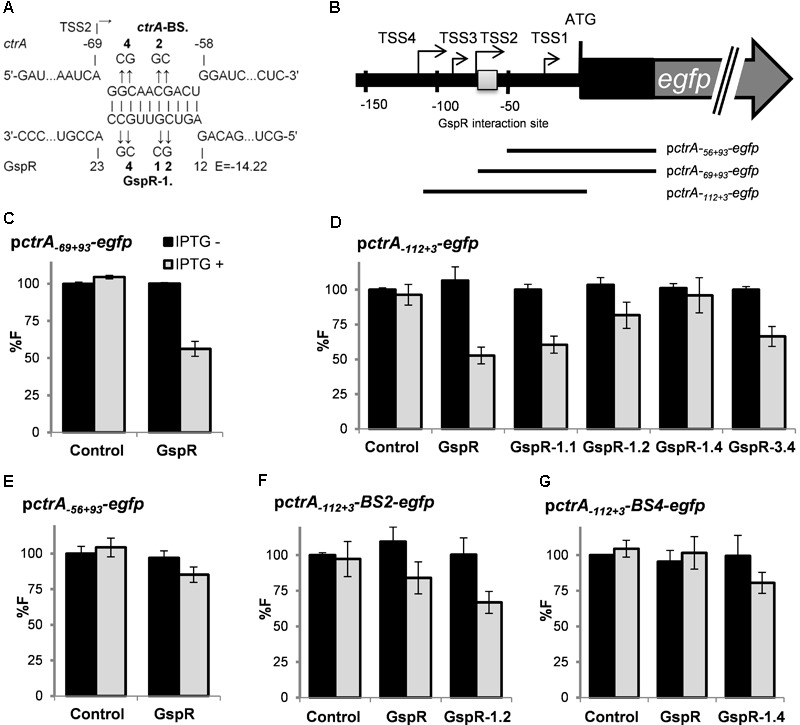
GspR SL1 represses *ctrA* post-transcriptionally. **(A)** Predicted duplexes between GspR and *ctrA* mRNA. The nucleotide exchanges in the predicted interaction regions of *ctrA* (*ctrA*-BS.2 and BS.4) and GspR (GspR 1.1, 1.2, 1.4, and 3.4) mRNAs are indicated in bold. **(B)** Schematic representations of the genomic regions and the fragments translationally fused to *egfp*. **(C–G)** Means of relative fluorescence intensity values of Sm2B2019DD co-transformed with overexpression plasmids carrying control *SmelC812, gspR*, or its mutant variants and the indicated *ctrA* translational fusion. Fluorescence measurements have been performed as described in **Figure 4**.

For the *ctrA* mRNA, continuous base pairing involving a 10-nt stretch of the target and GspR SL1 was predicted (**Figures 5A,B**). Multiple TSSs have been reported for *ctrA.* Two of these, TSS2 (**Figure 5B**) and TSS6 (position -291) are preceded by consensus promoter motifs and associated with strongly accumulating transcripts (Schlüter et al., 2013). The putative interaction sequence of *ctrA* mRNA with GspR SL1 starts 1 nt after TSS2 (**Figures 5A,B**). The post-transcriptional effect of GspR on *ctrA* expression was analyzed by different reporter constructs (**Figure 5B**). Compared to the control, *gspR* overexpression resulted in 44% and 47% decreased activity of the reporter constructs *ctrA*_-69+93_ (TSS2) and *ctrA*_-112+3_ (TSS4), respectively, both including the putative interaction site with GspR (**Figures 5C,D**). However, activity of reporter construct *ctrA*_-56+93_, which does not harbor the predicted interaction sequence, did not significantly change upon induced *gspR* overexpression, further supporting the prediction (**Figure 5E**). Moreover, 1, 2, and 4 nt changes in GspR SL1 (*gspR-1.1, gspR-1.2*, and *gspR-1.4*; **Figure 5A**) progressively mitigated the GspR-mediated repression of reporter expression in the same strain background and culture conditions previously used in the assays with wild-type GspR (**Figure 5D**). In contrast, mutations in GspR SL3 did not affect GspR-mediated repression of *ctrA* (**Figure 5D**). Concomitantly, introduction of 2 or 4 nt changes into the predicted binding site within the *ctrA* mRNA in the reporter constructs, leading to *ctrA*-BS.2 and *ctrA*-BS.4 (**Figure 5A**), also abolished the negative regulatory effect of GspR on reporter activity (**Figures 5F,G**). Combined compensatory mutations of *ctrA*-BS.2 or *ctrA*-BS.4 and GspR1.2 or GspR1.4, respectively, partially restored the regulation by GspR (**Figures 5F,G**). Altogether, these data validate *ctrA* mRNA as target of GspR and strongly suggest that this regulation is mediated by base pairing with complementary nucleotides within the SL1 single stranded region of GspR.

We also tested the GspR mutant variants GspR-1.4 and GspR-3.4 for their potential to repress growth of *S. meliloti* cultures. While overproduction of wild-type GspR or GspR-1.4 negatively affected growth, overproduction of GspR-3.4 or the control RNA SmelC812 did not significantly slow down growth (Supplementary Figure S4F). This indicates that the growth phenotype caused by elevated levels of GspR is associated with SL3, and thus involves regulation of other target mRNAs.

### CspA5 Enhances *ctrA* mRNA Translation Efficiency

**FIGURE 6:**
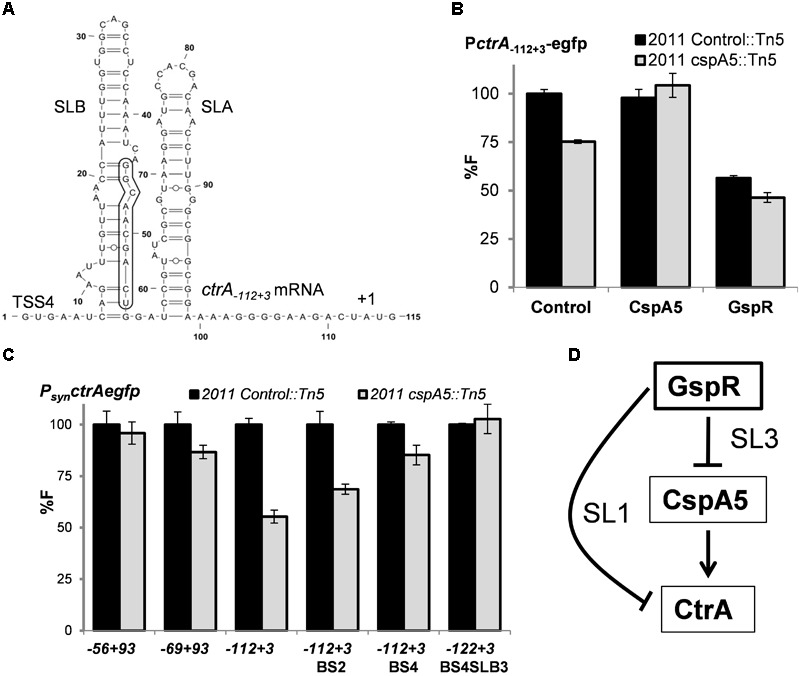
CspA5 facilitates *ctrA* translation. **(A)** RNAfold predicted secondary structure of *ctrA_-112+3_* mRNA transcripts exhibiting two stem loop (SL) structures. Nucleotide positions relative to the 5′-end of TSS4 are indicated. The 10-nt GspR interaction region on *ctrA* mRNA SLB is boxed. Means of relative fluorescence intensity values in the Rm2011 Control::Tn5 or Rm2011 *cspA5*::Tn5 background cells: **(B)** co-transformed with *ctrA_-112+3_* translational fusion and control plasmid pSRKGm, P_lac_*cspA5* or pSKGspR^+^ in the presence of IPTG; **(C)** transformed with the indicated *ctrA* native translational fusions or its mutant variants. Fluorescence measurements have been performed as described in **Figure 4**. **(D)** Schematic representation of the coherent feed forward loop circuit formed by the sRNA GspR and its *ctrA* and *cspA5* mRNA targets, which are negatively regulated through GspR SL1 and SL3, respectively.

CspA5 homologs act as nucleic acid chaperones, preventing the formation of mRNA secondary structures, thereby facilitating mRNA transcription and/or translation (Barria et al., 2013; Keto-Timonen et al., 2016). The *ctrA* mRNA transcribed from TSS4 is predicted to fold into a secondary structure formed by two stem loops (SLA and SLB, **Figure 6A**). We therefore wondered whether CspA5 post-transcriptionally influences *ctrA* expression.

Fluorescence values yielded by different *ctrA*-*egfp* translational fusions were compared in the background of a *S. meliloti cspA5*::mini-Tn*5* mutant and the control strain Rm2011 Control::Tn*5*, carrying a mini-Tn*5* insertion in the IGR between *sodB* and *SMc00107* (Schlüter et al., 2013). This strain has been reported not to be impaired in growth under several culture and stress conditions (Pobigaylo et al., 2006). The reporter construct *ctrA*_-112+3_ (TSS4) showed lower activity in cells lacking functional CspA5 than in control cells (**Figures 6B,C**). This phenotype was complemented by ectopic expression of full-length *cspA5* from the P_lac_ promoter (**Figure 6B**). Besides that, GspR overproduction resulted in a decrease in *ctrA-egfp* reporter activity in the *cspA5* mutant compared to the control strain, suggesting that both post-transcriptional mechanisms of control are not mutually exclusive (**Figure 6C**). This decrease in activity was not evident for reporter constructs encoding shorter portions of the *ctrA* mRNA (-69/+3 and -56/+3 relative to the *ctrA* start codon; **Figure 6C**). The predicted secondary RNA structures of these constructs lack the longer SLB of the *ctrA* mRNA (Supplementary Figures S7A,B), which may represent the inhibitory structure melted by CspA5. The *ctrA*_-112+3_ reporter construct carrying 2 or 4 nt changes in the SLB stem (*ctrA*-BS.2 and *ctrA*-BS.4) still showed less *ctrA-egfp* derived fluorescence in the *cspA5* mutant (**Figure 6C**). These constructs are predicted to fold similar to the ones with the native 5′-UTR sequence (Supplementary Figures S7C,D). However, the combination of the *ctrA*-BS.4 changes with three additional mutations in SLB, resulting in an altered predicted secondary structure (Supplementary Figure S7E), showed similar levels of reporter activity and was not affected by the *cspA5* mutation (**Figure 6C**). In summary, these results indicate that CspA5 positively regulates CtrA, probably by uncoiling SLB formed in the long *ctrA* 5′ UTR.

## Discussion

Cellular levels of master transcriptional regulators governing key physiological processes must be strictly controlled to ensure adaptation to environmental changes and bacterial survival. In α-proteobacteria, the master cell cycle regulator CtrA controls expression of more than a hundred genes and underlies multiple levels of regulation, which guarantee coordinated progression of fundamental cell cycle processes and adaptation to stress conditions. Regulatory mechanisms acting at the transcriptional level or influencing activity and levels of CtrA by phosphorylation and proteolytic degradation have been extensively studied (Domian et al., 1997; Reisenauer and Shapiro, 2002; Holtzendorff et al., 2004; Iniesta et al., 2006; Smith et al., 2014; Heinrich et al., 2016). In this work, we showed that the *trans*-sRNA GspR and the RNA chaperone CspA5 expand the diverse regulation of CtrA by mechanisms addressing the *ctrA* mRNA in *S. meliloti*. This additional level of regulation probably contributes to further control CtrA and thereby reinforces resilience and robustness of the mechanisms governing α-proteobacterial cell cycle progression in different growth phases and changing environments.

### GspR Targets and Functional Role

Compared to the wild type, loss of CtrA in *Rhodobacter capsulatus* led to the recent identification of 18 differentially expressed and so far functionally uncharacterized sRNAs (Grüll et al., 2017). Conversely, *trans*-sRNA mediated post-transcriptional modulation of genes related to cell cycle progression has previously been reported in *S. meliloti*. Here, the EcpR1 sRNA post-transcriptionally modulates expression of *dnaA* and *gcrA* (Robledo et al., 2015). The discovery of GspR to influence stability or translation of the *ctrA* mRNA adds this level of regulation to another master regulator of the complex circuitry governing cell cycle progression in *S. meliloti*. While EcpR1 is functionally conserved in several members of the Rhizobiales (Reinkensmeier et al., 2011; Robledo et al., 2015), GspR was only identified in species belonging to the *Sinorhizobium* genus. This suggests GspR-mediated regulation to have evolved as a specific adaptation within this genus. Moreover, cell growth arrest upon *gspR* overexpression was specifically observed in various *Sinorhizobium* strains, suggesting functional conservation. Although we were unable to detect significant cell growth or morphology phenotypes in a *gspR* deletion mutant, this mutant was moderately outcompeted by the wild type in fitness assays. Because of functional redundancies in regulatory mechanisms, i.e., the mechanisms controlling CtrA levels, bacterial sRNA knock-out mutants frequently lack strong phenotypes under experimental conditions (Storz et al., 2011).

*Sinorhizobium meliloti* is able to establish a root nodule symbiosis with leguminous host plants. In the course of this interaction, the bacteria invade the root nodules and terminally differentiate to polyploid nitrogen-fixing bacteroids (Oke and Long, 1999; Oldroyd et al., 2011). Several studies propose that CtrA depletion is an important feature in *S. meliloti* bacteroid differentiation, but the underlying mechanisms remain veiled (Penterman et al., 2014; Roux et al., 2014; Pini et al., 2015). While GspR was dispensable for a functional symbiosis, it cannot be ruled out that GspR contributes to fine-tune the establishment of this interaction. Supporting this possibility, a transcriptome study of individual root nodule zones detected high levels of GspR in the regions of plant cell infection and bacteroid differentiation, but not in the nitrogen-fixation zone containing mature bacteroids (Roux et al., 2014).

The functions of the two experimentally confirmed GspR targets, *ctrA* encoding a transcriptional regulator and *cspA5* coding for a non-specific RNA chaperone, suggest secondary downstream effects of GspR-mediated regulation. In agreement with this expectation, we identified members of the CtrA regulon (Pini et al., 2015) among the genes showing altered expression upon GspR overproduction (Supplementary Data Sheet S1). These include *btaA*, coding for a protein involved in cell envelope synthesis, and the motility genes *flaA, flaC*, and *flgG*; *btaA* being upregulated and the motility genes downregulated both under GspR overproduction and CtrA depletion conditions (Pini et al., 2015). Furthermore, the 5′-UTR of the *minC* mRNA, but not the coding region, was significantly less abundant in *gspR* overexpressing cells. In *E. coli*, MinC, MinD, and MinE act in concert to control formation and positioning of the FtsZ-ring, which drives septation (Cheng et al., 2007). In *S. meliloti, minCDE* is dispensable, but either *minC-minD* overexpression or disruption of *minE* cause severe growth phenotypes (Cheng and Walker, 1998). Interestingly, *minC* is repressed by CtrA, while the majority of genes was found to be activated by this regulator (Pini et al., 2015).

### Two Single-Stranded Loop Regions of GspR Bind Different mRNA Targets

The confirmed interaction regions of GspR with the *cspA5* and *ctrA* mRNAs reflect different mechanisms of inhibition. GspR SL3 seems to mediate canonical translational repression by targeting the translation initiation region in *cspA5* mRNA, whereas GspR SL1 most probably binds a region -69 to -58 nucleotides upstream of the start codon in the *ctrA* mRNA. Repression of translation at target sequences located upstream of the ribosome binding site has already been reported for other sRNA–mRNA pairs, e.g., GcvB-*gltl* and EcpR1-*gcrA* (Sharma et al., 2007; Robledo et al., 2015), but the underlying mechanisms are largely unknown. Two to four nucleotide changes in GspR SL1 were necessary to abolish its regulatory activity on *ctrA*, suggesting a high strength of sRNA-mRNA binding, similar to the EcpR1-*gcrA* pairing (Robledo et al., 2015).

While some sRNAs bind multiple targets through the same single-stranded domain (Balbontín et al., 2010; Robledo et al., 2015), others use distinct functional domains for multiple targeting (Overlöper et al., 2014; Feng et al., 2015). GspR matches the second group, with two confirmed targets pairing to SL1 and SL3 domains. Overproduction of GspR mutant variants carrying changes in SL1 or SL3 did not show off-target regulation, supporting both GspR stem loops as independent functional domains with respect to regulation of *ctrA* and *cspA5*. Intriguingly, northern and RNAseq data indicated two coexisting GspR RNAs: the full-length transcript and a shorter, less abundant variant lacking SL1 and therefore being unable to regulate *ctrA*. Different stabilities of the two GspR variants may influence the regulatory activity and specificity of GspR.

Although, *gspR* overexpression appeared to significantly reduce translation of the *ctrA* mRNA and both CtrA-depleted (Pini et al., 2015) and GspR-overproducing cells showed growth arrest phenotypes, these phenotypes cannot be associated to GspR-mediated regulation of *ctrA* through SL1. Our mutational studies link growth repression caused by GspR to SL3, which targets *cspA5* mRNA. However, a *cspA5* insertion mutant was not hampered in growth, indicating that GspR-mediated regulation of yet uncharacterized targets, individually or in combination, causes this phenotype.

### GspR Controls a Feed-Forward Loop Involving CspA5 and CtrA

Cold shock proteins have been reported to facilitate mRNA translation under different stress conditions, including cold, thereby contributing to bacterial adaptation to changing environments (Keto-Timonen et al., 2016). In this study, we revealed regulation of *ctrA* expression by CspA5 in *S. meliloti*. The role of CspAs has not been addressed yet in rhizobacteria and its homology to cold shock proteins does not necessarily imply that they have a role at low temperatures. Eight CspA homologs were found in *S. meliloti*: chromosomally encoded CspA1 to 5, and pSymA-encoded CspA6 to 8. Similarly, *E. coli* carries nine *csp* genes and at least four have to be deleted to generate a growth phenotype (Xia et al., 2001). Therefore, the lack of a growth phenotype of the *S. meliloti cspA5* mutant is not surprising.

Bacteria use different network motifs involving a variable number of regulators (Beisel and Storz, 2010). Recent studies in enterobacteria have stressed the role of sRNAs together with transcriptional factors in regulatory networks (Beisel and Storz, 2011; Papenfort et al., 2015). Feed-forward loops are composed of one regulator controlling a second regulator and a target gene controlled by both these regulators (Beisel and Storz, 2010). When both arms of the loop act jointly, the loop is classified as coherent. Here, we show that GspR sRNA post-transcriptionally represses the mRNAs of the cold shock chaperone homolog CspA5 and the cell cycle master regulator CtrA, which is post-transcriptionally controlled by CspA5 (**Figure 6D**), thus forming a coherent feed-forward loop. This regulatory circuit may enhance the negative effect of GspR on *ctrA* expression. On the one hand, it directly represses *ctrA* and, on the other hand, it negatively influences its activator CspA5. Intriguingly, the *cspA5* promoter region harbors a putative CtrA binding motif (Schlüter et al., 2013), but direct regulation has not been proved under the conditions tested. This may hint to a further link in this network. To the best of our knowledge, GspR is the first sRNA reported to post-transcriptionally control via separate seed-pairing domains both targets in a feed-forward loop.

## Author Contributions

AB, MR, J-PS, and JJ-Z conceived and designed the experiments. MR and J-PS performed the main experiments. J-PS, LL, UL, and SA carried out the transcriptome and proteome profiling. MR and AB analyzed the data. MR, AB, and JJ-Z wrote the paper.

## Conflict of Interest Statement

The authors declare that the research was conducted in the absence of any commercial or financial relationships that could be construed as a potential conflict of interest.

## References

[B1] Albaum S. P. Hahne H. Otto A. Haußmann U. Becher D. Poetsch A. (2011). A guide through the computational analysis of isotope-labeled mass spectrometry-based quantitative proteomics data: an application study. *Proteome Sci.* 9:30. 10.1186/1477-5956-9-30 21663690 PMC3142201

[B2] Albaum S. P. Neuweger H. Fränzel B. Lange S. Mertens D. Trötschel C. (2009). Qupe–a rich internet application to take a step forward in the analysis of mass spectrometry-based quantitative proteomics experiments. *Bioinformatics* 25 3128–3134. 10.1093/bioinformatics/btp568 19808875

[B3] Bahlawane C. McIntosh M. Krol E. Becker A. (2008). *Sinorhizobium meliloti* regulator MucR couples exopolysaccharide synthesis and motility. *Mol. Plant Microbe Interact.* 21 1498–1509. 10.1094/MPMI-21-11-1498 18842098

[B4] Balbontín R. Fiorini F. Figueroa-Bossi N. Casadesús J. Bossi L. (2010). Recognition of heptameric seed sequence underlies multi-target regulation by RybB small RNA in *Salmonella enterica*. *Mol. Microbiol.* 78 380–394. 10.1111/j.1365-2958.2010.07342.x 20979336

[B5] Barria C. Malecki M. Arraiano C. M. (2013). Bacterial adaptation to cold. *Microbiology* 159(Pt 12) 2437–2443. 10.1099/mic.0.052209-0 24068238

[B6] Baumgardt K. Charoenpanich P. McIntosh M. Schikora A. Stein E. Thalmann S. (2014). RNase E affects the expression of the acyl-homoserine lactone synthase gene sinI in *Sinorhizobium meliloti*. *J. Bacteriol.* 196 1435–1447. 10.1128/JB.01471-13 24488310 PMC3993346

[B7] Becker A. Overlöper A. Schlüter J. P. Reinkensmeier J. Robledo M. Giegerich R. (2014). Riboregulation in plant-associated α-proteobacteria. *RNA Biol.* 11 550–562. 10.4161/rna.29625 25003187 PMC4152362

[B8] Beisel C. L. Storz G. (2010). Base pairing small RNAs and their roles in global regulatory networks. *FEMS Microbiol. Rev.* 34 866–882. 10.1111/j.1574-6976.2010.00241.x 20662934 PMC2920360

[B9] Beisel C. L. Storz G. (2011). The base-pairing RNA spot 42 participates in a multioutput feedforward loop to help enact catabolite repression in *Escherichia coli*. *Mol. Cell* 41 286–297. 10.1016/j.molcel.2010.12.027 21292161 PMC3072601

[B10] Beringer J. E. (1974). R factor transfer in *Rhizobium leguminosarum*. *J. Gen. Microbiol.* 84 188–198. 10.1099/00221287-84-1-188 4612098

[B11] Biondi E. G. Reisinger S. J. Skerker J. M. Arif M. Perchuk B. S. Ryan K. R. (2006). Regulation of the bacterial cell cycle by an integrated genetic circuit. *Nature* 444 899–904. 10.1038/nature05321 17136100

[B12] Brilli M. Fondi M. Fani R. Mengoni A. Ferri L. Bazzicalupo M. (2010). The diversity and evolution of cell cycle regulation in alpha-proteobacteria: a comparative genomic analysis. *BMC Syst. Biol.* 4:52. 10.1186/1752-0509-4-52 20426835 PMC2877005

[B13] Cheng H. P. Walker G. C. (1998). Succinoglycan is required for initiation and elongation of infection threads during nodulation of alfalfa by *Rhizobium meliloti*. *J. Bacteriol.* 180 5183–5191. 9748453 10.1128/jb.180.19.5183-5191.1998 PMC107556

[B14] Cheng J. Sibley C. D. Zaheer R. Finan T. M. (2007). A *Sinorhizobium meliloti* minE mutant has an altered morphology and exhibits defects in legume symbiosis. *Microbiology* 153(Pt 2) 375–387. 10.1099/mic.0.2006/001362-0 17259609

[B15] Darty K. Denise A. Ponty Y. (2009). VARNA: interactive drawing and editing of the RNA secondary structure. *Bioinformatics* 25 1974–1975. 10.1093/bioinformatics/btp250 19398448 PMC2712331

[B16] Domian I. J. Quon K. C. Shapiro L. (1997). Cell type-specific phosphorylation and proteolysis of a transcriptional regulator controls the G1-to-S transition in a bacterial cell cycle. *Cell* 90 415–424. 10.1016/S0092-8674(00)80502-4 9267022

[B17] Dondrup M. Albaum S. P. Griebel T. Henckel K. Jünemann S. Kahlke T. (2009). EMMA 2–a MAGE-compliant system for the collaborative analysis and integration of microarray data. *BMC Bioinformatics* 10:50. 10.1186/1471-2105-10-50 19200358 PMC2645365

[B18] Feng L. Rutherford S. T. Papenfort K. Bagert J. D. van Kessel J. C. Tirrell D. A. (2015). A qrr noncoding RNA deploys four different regulatory mechanisms to optimize quorum-sensing dynamics. *Cell* 160 228–240. 10.1016/j.cell.2014.11.051 25579683 PMC4313533

[B19] Frage B. Döhlemann J. Robledo M. Lucena D. Sobetzko P. Graumann P. L. (2016). Spatiotemporal choreography of chromosome and megaplasmids in the *Sinorhizobium meliloti* cell cycle. *Mol. Microbiol.* 100 808–823. 10.1111/mmi.13351 26853523

[B20] Galibert F. Finan T. M. Long S. R. Puhler A. Abola P. Ampe F. (2001). The composite genome of the legume symbiont *Sinorhizobium meliloti*. *Science* 293 668–672. 10.1126/science.1060966 11474104

[B21] Giacomini A. Ollero F. J. Squartini A. Nuti M. P. (1994). Construction of multipurpose gene cartridges based on a novel synthetic promoter for high-level gene expression in gram-negative bacteria. *Gene* 144 17–24. 10.1016/0378-1119(94)90197-X 8026755

[B22] Gottesman S. Storz G. (2011). Bacterial small RNA regulators: versatile roles and rapidly evolving variations. *Cold Spring Harb. Perspect. Biol.* 3:a003798. 10.1101/cshperspect.a003798 20980440 PMC3225950

[B23] Gruber A. R. Lorenz R. Bernhart S. H. Neuböck R. Hofacker I. L. (2008). The Vienna RNA website. *Nucleic Acids Res.* 36 W70–W74. 10.1093/nar/gkn188 18424795 PMC2447809

[B24] Grüll M. P. Peña-Castillo L. Mulligan M. E. Lang A. S. (2017). Genome-wide identification and characterization of small RNAs in *Rhodobacter capsulatus* and identification of small RNAs affected by loss of the response regulator CtrA. *RNA Biol.* 14 914–925. 10.1080/15476286.2017.1306175 28296577 PMC5546546

[B25] Heinrich K. Sobetzko P. Jonas K. (2016). A Kinase-Phosphatase switch transduces environmental information into a bacterial cell cycle circuit. *PLoS Genet.* 12:e1006522. 10.1371/journal.pgen.1006522 27941972 PMC5189948

[B26] Holtzendorff J. Hung D. Brende P. Reisenauer A. Viollier P. H. McAdams H. H. (2004). Oscillating global regulators control the genetic circuit driving a bacterial cell cycle. *Science* 304 983–987. 10.1126/science.1095191 15087506

[B27] Iniesta A. A. McGrath P. T. Reisenauer A. McAdams H. H. Shapiro L. (2006). A phospho-signaling pathway controls the localization and activity of a protease complex critical for bacterial cell cycle progression. *Proc. Natl. Acad. Sci. U.S.A.* 103 10935–10940. 10.1073/pnas.0604554103 16829582 PMC1544152

[B28] Jiménez-Zurdo J. I. Robledo M. (2015). Unraveling the universe of small RNA regulators in the legume symbiont *Sinorhizobium meliloti*. *Symbiosis* 67 43–54. 10.1007/s13199-015-0345-z

[B29] Jiménez-Zurdo J. I. Valverde C. Becker A. (2013). Insights into the noncoding RNome of nitrogen-fixing endosymbiotic α-proteobacteria. *Mol. Plant Microbe Interact.* 26 160–167. 10.1094/MPMI-07-12-0186-CR 22991999

[B30] Keto-Timonen R. Hietala N. Palonen E. Hakakorpi A. Lindström M. Korkeala H. (2016). Cold shock proteins: a minireview with special emphasis on Csp-family of enteropathogenic *Yersinia*. *Front. Microbiol.* 7:1151. 10.3389/fmicb.2016.01151 27499753 PMC4956666

[B31] McAdams H. H. Shapiro L. (2009). System-level design of bacterial cell cycle control. *FEBS Lett.* 583 3984–3991. 10.1016/j.febslet.2009.09.030 19766635 PMC2795017

[B32] Oke V. Long S. R. (1999). Bacteroid formation in the *Rhizobium*-legume symbiosis. *Curr. Opin. Microbiol.* 2 641–646. 10.1016/S1369-5274(99)00035-1 10607628

[B33] Oldroyd G. E. Murray J. D. Poole P. S. Downie J. A. (2011). The rules of engagement in the legume-rhizobial symbiosis. *Annu. Rev. Genet.* 45 119–144. 10.1146/annurev-genet-110410-132549 21838550

[B34] Overlöper A. Kraus A. Gurski R. Wright P. R. Georg J. Hess W. R. (2014). Two separate modules of the conserved regulatory RNA AbcR1 address multiple target mRNAs in and outside of the translation initiation region. *RNA Biol.* 11 624–640. 10.4161/rna.29145 24921646 PMC4152367

[B35] Papenfort K. Espinosa E. Casadesús J. Vogel J. (2015). Small RNA-based feedforward loop with AND-gate logic regulates extrachromosomal DNA transfer in *Salmonella*. *Proc. Natl. Acad. Sci. U.S.A.* 112 E4772–E4781. 10.1073/pnas.1507825112 26307765 PMC4553797

[B36] Penterman J. Abo R. P. De Nisco N. J. Arnold M. F. Longhi R. Zanda M. (2014). Host plant peptides elicit a transcriptional response to control the *Sinorhizobium meliloti* cell cycle during symbiosis. *Proc. Natl. Acad. Sci. U.S.A.* 111 3561–3566. 10.1073/pnas.1400450111 24501120 PMC3948309

[B37] Perkins D. N. Pappin D. J. Creasy D. M. Cottrell J. S. (1999). Probability-based protein identification by searching sequence databases using mass spectrometry data. *Electrophoresis* 20 3551–3567. 10.1002/(SICI)1522-2683(19991201)20:18<3551::AID-ELPS3551>3.0.CO;2-2 10612281

[B38] Pini F. De Nisco N. J. Ferri L. Penterman J. Fioravanti A. Brilli M. (2015). Cell cycle control by the master regulator CtrA in *Sinorhizobium meliloti*. *PLoS Genet.* 11:e1005232. 10.1371/journal.pgen.1005232 25978424 PMC4433202

[B39] Pobigaylo N. Wetter D. Szymczak S. Schiller U. Kurtz S. Meyer F. (2006). Construction of a large signature-tagged mini-Tn5 transposon library and its application to mutagenesis of *Sinorhizobium meliloti*. *Appl. Environ. Microbiol.* 72 4329–4337. 10.1128/AEM.03072-05 16751548 PMC1489598

[B40] Quon K. C. Marczynski G. T. Shapiro L. (1996). Cell cycle control by an essential bacterial two-component signal transduction protein. *Cell* 84 83–93. 10.1016/S0092-8674(00)80995-2 8548829

[B41] Reidegeld K. A. Eisenacher M. Kohl M. Chamrad D. Körting G. Blüggel M. (2008). An easy-to-use decoy database builder software tool, implementing different decoy strategies for false discovery rate calculation in automated MS/MS protein identifications. *Proteomics* 8 1129–1137. 10.1002/pmic.200701073 18338823

[B42] Reinkensmeier R. Schlüter J. P. Giegerich R. Becker A. (2011). Conservation and occurrence of trans-encoded sRNAs in the Rhizobiales. *Genes* 2 925–956. 10.3390/genes2040925 24710299 PMC3927594

[B43] Reisenauer A. Shapiro L. (2002). DNA methylation affects the cell cycle transcription of the CtrA global regulator in *Caulobacter*. *EMBO J.* 21 4969–4977. 10.1093/emboj/cdf490 12234936 PMC126286

[B44] Robertsen B. K. Aman P. Darvill A. G. McNeil M. Albersheim P. (1981). Host-Symbiont interactions: V. The structure of acidic extracellular polysaccharides secreted by *Rhizobium leguminosarum* and *Rhizobium trifolii*. *Plant Physiol.* 67 389–400. 10.1104/pp.67.3.389 16661681 PMC425692

[B45] Robledo M. Frage B. Wright P. R. Becker A. (2015). A stress-induced small RNA modulates alpha-rhizobial cell cycle progression. *PLoS Genet.* 11:e1005153. 10.1371/journal.pgen.1005153 25923724 PMC4414408

[B46] Robledo M. García-Tomsig N. I. Jiménez-Zurdo J. I. (2018). Primary characterization of small RNAs in symbiotic nitrogen-fixing bacteria. *Methods Mol. Biol.* 1734 277–295. 10.1007/978-1-4939-7604-1_22 29288462

[B47] Robledo M. Peregrina A. Millán V. García-Tomsig N. I. Torres-Quesada O. Mateos P. F. (2017). A conserved α-proteobacterial small RNA contributes to osmoadaptation and symbiotic efficiency of rhizobia on legume roots. *Environ. Microbiol.* 19 2661–2680. 10.1111/1462-2920.13757 28401641

[B48] Roux B. Rodde N. Jardinaud M. F. Timmers T. Sauviac L. Cottret L. (2014). An integrated analysis of plant and bacterial gene expression in symbiotic root nodules using laser-capture microdissection coupled to RNA sequencing. *Plant J.* 77 817–837. 10.1111/tpj.12442 24483147

[B49] Sanselicio S. Bergé M. Théraulaz L. Radhakrishnan S. K. Viollier P. H. (2015). Topological control of the *Caulobacter* cell cycle circuitry by a polarized single-domain PAS protein. *Nat. Commun.* 6:7005. 10.1038/ncomms8005 25952018 PMC4432633

[B50] Schlüter J. P. Reinkensmeier J. Barnett M. J. Lang C. Krol E. Giegerich R. (2013). Global mapping of transcription start sites and promoter motifs in the symbiotic α-proteobacterium *Sinorhizobium meliloti* 1021. *BMC Genomics* 14:156. 10.1186/1471-2164-14-156 23497287 PMC3616915

[B51] Schlüter J. P. Reinkensmeier J. Daschkey S. Evguenieva-Hackenberg E. Janssen S. Jänicke S. (2010). A genome-wide survey of sRNAs in the symbiotic nitrogen-fixing alpha-proteobacterium *Sinorhizobium meliloti*. *BMC Genomics* 11:245. 10.1186/1471-2164-11-245 20398411 PMC2873474

[B52] Sharma C. M. Darfeuille F. Plantinga T. H. Vogel J. (2007). A small RNA regulates multiple ABC transporter mRNAs by targeting C/A-rich elements inside and upstream of ribosome-binding sites. *Genes Dev.* 21 2804–2817. 10.1101/gad.447207 17974919 PMC2045133

[B53] Smith S. C. Joshi K. K. Zik J. J. Trinh K. Kamajaya A. Chien P. (2014). Cell cycle-dependent adaptor complex for ClpXP-mediated proteolysis directly integrates phosphorylation and second messenger signals. *Proc. Natl. Acad. Sci. U.S.A.* 111 14229–14234. 10.1073/pnas.1407862111 25197043 PMC4191750

[B54] Sobrero P. Schlüter J. P. Lanner U. Schlosser A. Becker A. Valverde C. (2012). Quantitative proteomic analysis of the Hfq-regulon in *Sinorhizobium meliloti* 2011. *PLoS One* 7:e48494. 10.1371/journal.pone.0048494 23119037 PMC3484140

[B55] Storz G. Vogel J. Wassarman K. M. (2011). Regulation by small RNAs in bacteria: expanding frontiers. *Mol. Cell* 43 880–891. 10.1016/j.molcel.2011.08.022 21925377 PMC3176440

[B56] Torres-Quesada O. Millán V. Nisa-Martínez R. Bardou F. Crespi M. Toro N. (2013). Independent activity of the homologous small regulatory RNAs AbcR1 and AbcR2 in the legume symbiont *Sinorhizobium meliloti*. *PLoS One* 8:e68147. 10.1371/journal.pone.0068147 23869210 PMC3712013

[B57] Torres-Quesada O. Reinkensmeier J. Schlüter J. P. Robledo M. Peregrina A. Giegerich R. (2014). Genome-wide profiling of Hfq-binding RNAs uncovers extensive post-transcriptional rewiring of major stress response and symbiotic regulons in *Sinorhizobium meliloti*. *RNA Biol.* 11 563–579. 10.4161/rna.28239 24786641 PMC4152363

[B58] Waters L. S. Storz G. (2009). Regulatory RNAs in bacteria. *Cell* 136 615–628. 10.1016/j.cell.2009.01.043 19239884 PMC3132550

[B59] Wright P. R. Georg J. Mann M. Sorescu D. A. Richter A. S. Lott S. (2014). CopraRNA and IntaRNA: predicting small RNA targets, networks and interaction domains. *Nucleic Acids Res.* 42 W119–W123. 10.1093/nar/gku359 24838564 PMC4086077

[B60] Xia B. Ke H. Inouye M. (2001). Acquirement of cold sensitivity by quadruple deletion of the cspA family and its suppression by PNPase S1 domain in *Escherichia coli*. *Mol. Microbiol.* 40 179–188. 10.1046/j.1365-2958.2001.02372.x 11298285

[B61] Zhan H. J. Lee C. C. Leigh J. A. (1991). Induction of the second exopolysaccharide (EPSb) in *Rhizobium meliloti* SU47 by low phosphate concentrations. *J. Bacteriol.* 173 7391–7394. 10.1128/jb.173.22.7391-7394.1991 1938929 PMC209250

[B62] Zhang M. Z. Sun Z. C. Fu X. R. Nan F. F. Fan Q. X. Wu X. A. (2009). Analysis of serum proteome profiles of non-Hodgkin lymphoma for biomarker identification. *J. Proteomics* 72 952–959. 10.1016/j.jprot.2009.03.009 19361584

