# Supplementary material for: An sRNA and Cold Shock Protein Homolog-Based Feedforward Loop Post-transcriptionally Controls Cell Cycle Master Regulator CtrA
Source: Front Microbiol. 2018 Apr 24;9:763. doi: 10.3389/fmicb.2018.00763 (PMC5928217; doi:10.3389/fmicb.2018.00763)
Supplement: Supplementary file 1 [file Table_1.DOCX]

**Table S1.** Bacterial strains and plasmids used in this study.

| Strain/Plasmid | Relevant Characteristics | Source |
| --- | --- | --- |
| *S. meliloti* |  |  |
| Rm2011 | Nx^r^, Sm^r^, *expR*^-^; Sm resistant derivative of *S. meliloti* SU47. Wt | (1) |
| Rm2011DD | Rm2011 *gspR-smelC776* double deletion mutant, markerless. | This work |
| Rm2011DD*ecpR1* | Rm2011DD *ecpR1* triple deletion mutant, markerless. | This work |
| Rm2011*mcherry* | 2011 tagged with mCherry by pKOSm single chromosomal integration | (2) |
| Rm2011*egfp* | 2011 tagged with egpf by pKOSe single chromosomal integration | (2) |
| Rm2011DD*mcherry* | 2011DD tagged with mCherry by pKOSe single chromosomal integration | This work |
| Rm2011*hfq* | *hfq* insertion mutant, Gm^r^ | (3) |
| Rm2011*rne675* | 2011 carrying pK18mobII inserted in the 675th codon of *rne*, Km^r^ | (4) |
| 2011mTn5STM.4.03.D09 | 2011 derivative with mini-Tn5 inserted in Smc03844 Km^r^ | (5) |
| 2011mTn5STM.1.12.B07 | 2011 derivative with mini-Tn5 between *sodB* and Smc001071 Km^r^ |  |
| 2011mTn5STM.1.08.B05 | 2011 derivative with mini-Tn5 inserted in *cspA5* Km^r^ (2011*cspA5*::Tn5) | (5) |
| 1021.G1PELR12E4 | Rm1021 carrying a single integration plasmid with a *Smc03845*fragment | (6) |
| Sm2B2019 | Nx^r^, Sm^r^, *expR*^-^; Rm2011 derivative ∆*sinRI*, markerless.  Referred to as 2019 in this study. | M. McIntosh |
| Sm2B2019DD | 2019 *gspR-smelC776* double deletion mutant, markerless. | This work |
| *S. medicae* WSM419 | Acid^t^ , sardinian isolate; Nx^r^, Cm^r^ | (7) |
| *S. fredii* NGR234 | Broad-host-range bacterium isolated from *Lablab purpureus*, Rf^r^ | (8) |
| *A. tumefaciens* C58 | Wild-type, isolated from a cherry tree (Prunus) tumor Nx^r^ | (9) |
| *E. coli* |  |  |
| DH5α | F– *endA1 supE44 thi-1*l*-recA1 gyrA96 relA1 deoR*D *U169* | (10) |
| S17-1 | *E. coli* 294 Thi RP4-2-Tc::Mu-Km::Tn7 | (11) |
| Plasmids |  |  |
| pBBegfp | pBBR1MCS-2 derivative for promoter eGFP fusions; Km^r^ | (12) |
| pP*_gspR_*-egfp | pBBegfp with *gspR* promoter-*egfp* transcriptional fusion | This work |
| pP*_smelC776_*-egfp | pBBegfp with *smelC776* | This work |
| pK18*mobsacB* | Suicide plasmid in *S. meliloti*, *sacB*, *oriV*, Km^r^ | (13) |
| pKDD775-6 | pK18*mobsacB* with *gspR* flanking regions for gene SOEing | This work |
| pSRKKm | pBBR1MCS-2 derivative with a Plac promoter, *lacIq*, *lacZa*^+^, Km^r^ | (14) |
| pSKControl*^+^* | pSRKKm carrying the *smel812* coding sequence fused to *sinR*-P*_sinI_* | (2) |
| pSKGspR^+^ | pSRKKm carrying the *gspR* coding sequence fused to *sinR*-P*_sinI_* | This work |
| pSKSmelC776^+^ | pSRKKm carrying the *smelC776* coding sequence fused to *sinR*-P*_sinI_* | This work |
| pSKGspR-1.1^+^ | pSKGspR^+^ carrying 1 nt change in GspR SL1 | This work |
| pSKGspR-1.2^+^ | pSKGspR^+^ carrying 2 nt changes in GspR SL1 | This work |
| pSKGspR-1.4^+^ | pSKGspR^+^ carrying 4 nt changes in GspR SL1 | This work |
| pSKGspR-3.4^+^ | pSKGspR^+^ carrying 4 nt changes in GspR SL3 | This work |
| pSRKGm | pBBR1MCS-2 derivative with a Plac promoter, *lacIq*, *lacZa*^+^, Gm^r^ | (14) |
| pSGControl*^+^* | pSRKGm with *smel812* coding sequence fused to *sinR*-P*_sinI_* | (2) |
| pSGGspR*^+^* | pSRKGm with the *gspR* coding sequence fused to *sinR*-P*_sinI_* | This work |
| pSGSmelC776*^+^* | pSRKGm with the SmelC776 coding sequence fused to *sinR*-P*_sinI_* | This work |
| pSGCR01029*^+^* | pSRKGm with the SmelCR01029 coding sequence fused to *sinR*-P*_sinI_* | This work |
| pSGCR01763*^+^* | pSRKGm with the SmelR01763 coding sequence fused to *sinR*-P*_sinI_* | This work |
| pSGSmelC045*^+^* | pSRKGm with the SmelC045 coding sequence fused to *sinR*-P*_sinI_* | This work |
| P_lac_*cspA5* | pSRKGm carrying the CspA5 coding sequence | This work |
| pR_EGFP | Reporter fusion plasmid for cloning of sRNA targets, Tc^r^, Ap^r^ | (15) |
| p*gcrA_-122+3_-egfp* | pR_EGFP with the *gcrA::egfp* translational fusion from TSS (-122) | (2) |
| p*SMc02819*_-136+99_-*egfp* | pR_EGFP with the SMc02819*::egfp* translational fusion | This work |
| p*cspA5_-53+45_-egfp* | pR_EGFP with the *cspA5*::*egfp* translational fusion | This work |
| p*cspA5_-_BS-egfp* | p*cspA5_-53+45_-egfp* with 2 nt compensatory changes in *gspR* interaction region | This work |
| p*ctrA_-56+93_-egfp* | pR_EGFP with the *ctrA::egfp* translational fusion from -56 | This work |
| p*ctrA_-112+3_-egfp* | pR_EGFP with the *ctrA::egfp* translational fusion from TSS4 | (2) |
| p*ctrA_-_BS2-egfp* | p*ctrA_-112+3_-egfp* with 2 nt compensatory changes in *gspR* interaction region | This work |
| p*ctrA_-_BS4-egfp* | p*ctrA_-112+3_-egfp* with 4 nt compensatory changes in *gspR* interaction region | This work |
| p*ctrA_-_BS4SLB3-egfp* | p*ctrA_-112+3_-egfp* with 7 nt conformational changes in SLB | This work |

**References**

1. Casse F, Boucher, C., Julliot, J.S., Michel, M., and Denarie, J. 1979. Identification and characterization of large plasmids in *Rhizobium meliloti* using agarose-gel electorphoresis. J Gen Microbiol 113:229–242.

2. Robledo M, Frage B, Wright PR, Becker A. 2015. A stress-induced small RNA modulates alpha-rhizobial cell cycle progression. PLoS Genet 11:e1005153.

3. Voss B, Holscher M, Baumgarth B, Kalbfleisch A, Kaya C, Hess WR, Becker A, Evguenieva-Hackenberg E. 2009. Expression of small RNAs in Rhizobiales and protection of a small RNA and its degradation products by Hfq in *Sinorhizobium meliloti*. Biochem Biophys Res Commun 390:331-6.

4. Baumgardt K, Charoenpanich P, McIntosh M, Schikora A, Stein E, Thalmann S, Kogel KH, Klug G, Becker A, Evguenieva-Hackenberg E. 2014. RNase E affects the expression of the acyl-homoserine lactone synthase gene *sinI* in *Sinorhizobium meliloti*. J Bacteriol 196:1435-47.

5. Pobigaylo N, Wetter D, Szymczak S, Schiller U, Kurtz S, Meyer F, Nattkemper TW, Becker A. 2006. Construction of a large signature-tagged mini-Tn5 transposon library and its application to mutagenesis of *Sinorhizobium meliloti*. Appl Environ Microbiol 72:4329-37.

6. Bahlawane C, McIntosh M, Krol E, Becker A. 2008. *Sinorhizobium meliloti* regulator MucR couples exopolysaccharide synthesis and motility. Mol Plant Microbe Interact 21:1498-509.

7. Howieson JGaE, M. A. 1986. Acid tolerance in the *Rhizobium meliloti–Medicago* symbiosis. Aust J Agric Res 37:55–64.

8. Jarvis BD, Downer HL, Young JP. 1992. Phylogeny of fast-growing soybean-nodulating rhizobia support synonymy of *Sinorhizobium* and *Rhizobium* and assignment to *Rhizobium fredii*. Int J Syst Bacteriol 42:93-6.

9. Hamilton RH, Fall MZ. 1971. The loss of tumor-initiating ability in *Agrobacterium tumefaciens* by incubation at high temperature. Experientia 27:229-30.

10. Grant SG, Jessee J, Bloom FR, Hanahan D. 1990. Differential plasmid rescue from transgenic mouse DNAs into *Escherichia coli* methylation-restriction mutants. Proc Natl Acad Sci U S A 87:4645-9.

11. Simon R, Priefer U, Pühler A. 1983. A Broad Host Range Mobilization System for *In Vivo* Genetic Engineering: Transposon Mutagenesis in Gram Negative Bacteria. Nature Biotechnology 1:784-791.

12. Robledo M, Peregrina A, Millán V, García-Tomsig NI, Torres-Quesada O, Mateos PF, Becker A, Jiménez-Zurdo JI. 2017. A conserved α-proteobacterial small RNA contributes to osmoadaptation and symbiotic efficiency of rhizobia on legume roots. Environ Microbiol doi:10.1111/1462-2920.13757.

13. Schafer A, Tauch A, Jager W, Kalinowski J, Thierbach G, Puhler A. 1994. Small mobilizable multi-purpose cloning vectors derived from the *Escherichia coli* plasmids pK18 and pK19: selection of defined deletions in the chromosome of *Corynebacterium glutamicum*. Gene 145:69-73.

14. Khan SR, Gaines J, Roop RM, Farrand SK. 2008. Broad-host-range expression vectors with tightly regulated promoters and their use to examine the influence of TraR and TraM expression on Ti plasmid quorum sensing. Appl Environ Microbiol 74:5053-62.

15. Torres-Quesada O, Millán V, Nisa-Martínez R, Bardou F, Crespi M, Toro N, Jiménez-Zurdo JI. 2013. Independent activity of the homologous small regulatory RNAs AbcR1 and AbcR2 in the legume symbiont *Sinorhizobium meliloti*. PLoS One 8:e68147.
