# Supplementary material for: An sRNA and Cold Shock Protein Homolog-Based Feedforward Loop Post-transcriptionally Controls Cell Cycle Master Regulator CtrA
Source: Front Microbiol. 2018 Apr 24;9:763. doi: 10.3389/fmicb.2018.00763 (PMC5928217; doi:10.3389/fmicb.2018.00763)
Supplement: Supplementary file 2 [file Table_2.DOCX]

**Table S2.** Oligonucleotides used in this study.

| Primer name | Sequence (5´ -> 3´) | Use in this study ssstudystudy |
| --- | --- | --- |
| RS1.775i2 | GTTCGAGAACCTCCCGCAGCATGAC | Northern probes |
| RS2.775c2 | TCGAAGTTACCGGACCGAACCAGCC |  |
| RS3.776c2 | GGCTGGTTCGGTCCGGTAACTTCGA |  |
| RS4.7762 | CCGCCGCAAGCGAGGCGGAA |  |
| SpeI _775_TF-188 | attaACTAGTCGTAGTGACGTGCTTCGCCTT | Construction of promoter-*egfp* transcriptional fusions |
| XbaI_R775TF +29 | tctgTCTAGAATACGGTGGCAACGACTCTG |  |
| SpeI_776TF-229 | atgcACTAGTGGCAAGGAGATCGCCTATTGG |  |
| XbaI_F776TF +8 | tctgTCTAGAGGAACGTTTCCTGCGTCCTC |  |
| DDLeftFwdBamH1 | tctgGGATCCCAAGGCACTGGCGTTCATAC | Construction of pKDD775-6 and deletion confirmation |
| DDLeftRev | ATCGTGTGAGGCACACTGTC |  |
| DDRightFwdOvExt | GACAGTGTGCCTCACACGATCTGCGTCCTCAGAGCTTTCC |  |
| DDRightRevHindIII | tctgAAGCTTCCAACAATCCGACGTCTCAC |  |
| DD_Test_Fwd | GGCTGGCTGATGTCCATACC |  |
| DD_Test_Rev | ACGAGGTCACCTACGTCAAC |  |
| *sinR*_NdeIF | gccaCATATGGCTAATCAACAGGCTGTC | Construction of the sRNA overexpression plasmids |
| TSS3_28bp_b_*sinIR* | GTAGCGATGCTGTCAGGCTC |  |
| SmelC775_fwd | GAGCCTGACAGCATCGCTACCGCTGCCGGACAGAGTCGTTG |  |
| SmelC775_ XbaI_rev | tctgTCTAGAAGGAAACGTTCCGCCTCGCTTG |  |
| SmelC776_fwd | GAGCCTGACAGCATCGCTACAACGTTCCGCCTCGCTTG |  |
| SmelC776_XbaI_rev | tctgTCTAGAGTTCTCGAACGCAGACATGG |  |
| SmelCR1763_fwd | GAGCCTGACAGCATCGCTAC_TTAACAACGCGGCCGAGCAATG |  |
| SmelCR1763_XbaI_rev | tctg TCTAGATTGCCGGCCAGATGAAACGC |  |
| SmelC045_fwd | GAGCCTGACAGCATCGCTAC_ATGCCTGTGCTCGCTATCCATTTC |  |
| SmelC045_XbaI_rev | tctgTCTAGACTGTGCTTGCGCCGAAAGTG |  |
| SmelCR1029_fwd | GAGCCTGACAGCATCGCTACGCAATCGATCTCTTTTGCGGGCATTTGCAG |  |
| SmelCR1029_XbaI_rev | tctgTCTAGACCTCCTGTTGTTGCGGCAGAGATTAGC |  |
| NdeIcspAF TGACAGGACATCTTCGAAAG | gctgCATATGGCTGACAGGACATCTTCGAAAG | Construction of P_lac_*cspA5* |
| XbaIcspAOER TAATTCTAGATCAGTGCGACCGCGTGGTCG | taatTCTAGATCAGTGCGACCGCGTGGTCG |  |
| C775KO1.1F | GCTACGCTGCCGGACAGAGTCCTTGCCACCGTATGTCCGGCCA | Construction of GspR mutant variants |
| C775KO1.1R | GGACTCTGTCCGGCAGCGTAGC |  |
| C775KO1.2F | GCTACGCTGCCGGACAGAGTGCTTGCCACCGTATGTCCGGCCA |  |
| C775KO1.2R | GCACTCTGTCCGGCAGCGTAGC |  |
| C775KO1.4F | GCTACGCTGCCGGACAGAGTGCTTCGCACCGTATGTCCGGCCAGCCG |  |
| C775KO1.4R | CGAAGCACTCTGTCCGGCAGCGTAGC |  |
| C775KO3.4F | CCGGTAACTTCGAACCCTCAGGGGGAGCCCCTCGATGCTGCCGGG |  |
| C775KO3.4R | CCCCCTGAGGGTTCGAAGTTACCGG |  |
| Smc02819-136_F | ccggGGATCCGGGAAAGGCAATCGCAGCTTGT | Construction of the 5´UTR-*egfp* fusions |
| Smc02819+99 _R | cacgGCTAGCGCTCGCGGAACTCTCCTGAGCC |  |
| BcspA5-53F | agacGGATCCATAAGAATCAGTTAACCGTATC |  |
| NcspA5+45R | gtagGCTAGCTTCGCCATTATGGATGACGTCTT |  |
| cspA5KO3.2F | AGTGTTGCGCGTAACGAGGGCGTGCGAGAATGGCTGACAGGA |  |
| cspA5KO3.2R | CGCCCTCGTTACGCGCAACACT |  |
| BHI5UTRctrA56_F | gactGGATCCGTATCGCGTAAGG |  |
| 5UTRctrA96Nh_R | tgcaGCTAGCGAGATCTGTGGTGTAGACGT |  |
| ctrAKO2F | GGCAGCCTCCAAATCAGGCAAGCACTGGATCCGTATCGCGTAA |  |
| ctrAKO2R | GCTTGCCTGATTTGGAGGCTGCC |  |
| ctrAKO1.4F | GGCAGCCTCCAAATCAGCGAAGCACTGGATCCGTATCGCGTAA |  |
| ctrAKO1.4R | GCTTCGCTGATTTGGAGGCTGCC |  |
| ctrAKO sub1.3.4F | GAATTTGTTAACCATTTGGTGCGAGGCTCCAAATCAGCGAAGCACT |  |
| ctrAKOsub1.3R CCTCGCACCAAATGGTTAACAAATTC | CCTCGCACCAAATGGTTAACAAATTC |  |
| PCR 1 | CGGGCCTCTTCGCTATT | Sequencing |
| PCR 2 | TTAGCTCACTCATTAGG |  |
| *Egfp*-139_rev | GATGAACTTCAGGGTCAGCTTG |  |

Small case indicates linker regions and restriction site sequences are underlined.
