# Supplementary material for: An sRNA and Cold Shock Protein Homolog-Based Feedforward Loop Post-transcriptionally Controls Cell Cycle Master Regulator CtrA
Source: Front Microbiol. 2018 Apr 24;9:763. doi: 10.3389/fmicb.2018.00763 (PMC5928217; doi:10.3389/fmicb.2018.00763)
Supplement: Supplementary file 4 [file Image_1.PDF]

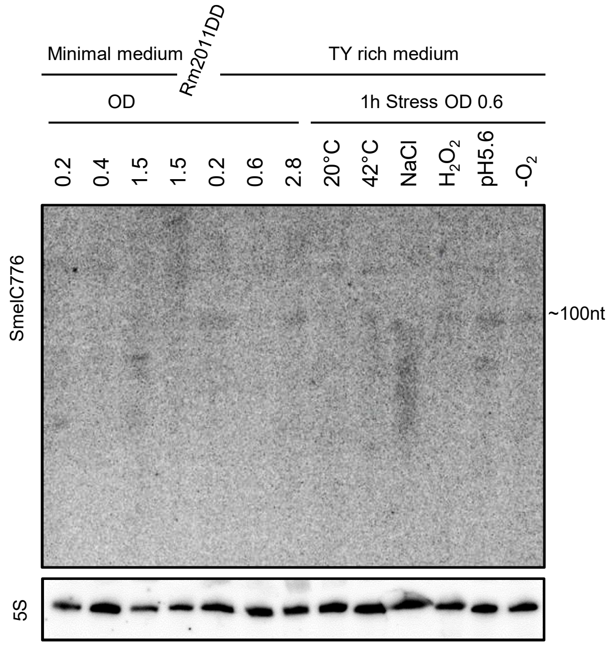

Figure S1. SmelC776 transcript is barely detected. Northern blot was performed as in Figure 2A using a SmelC776-oligonucleotide probe.

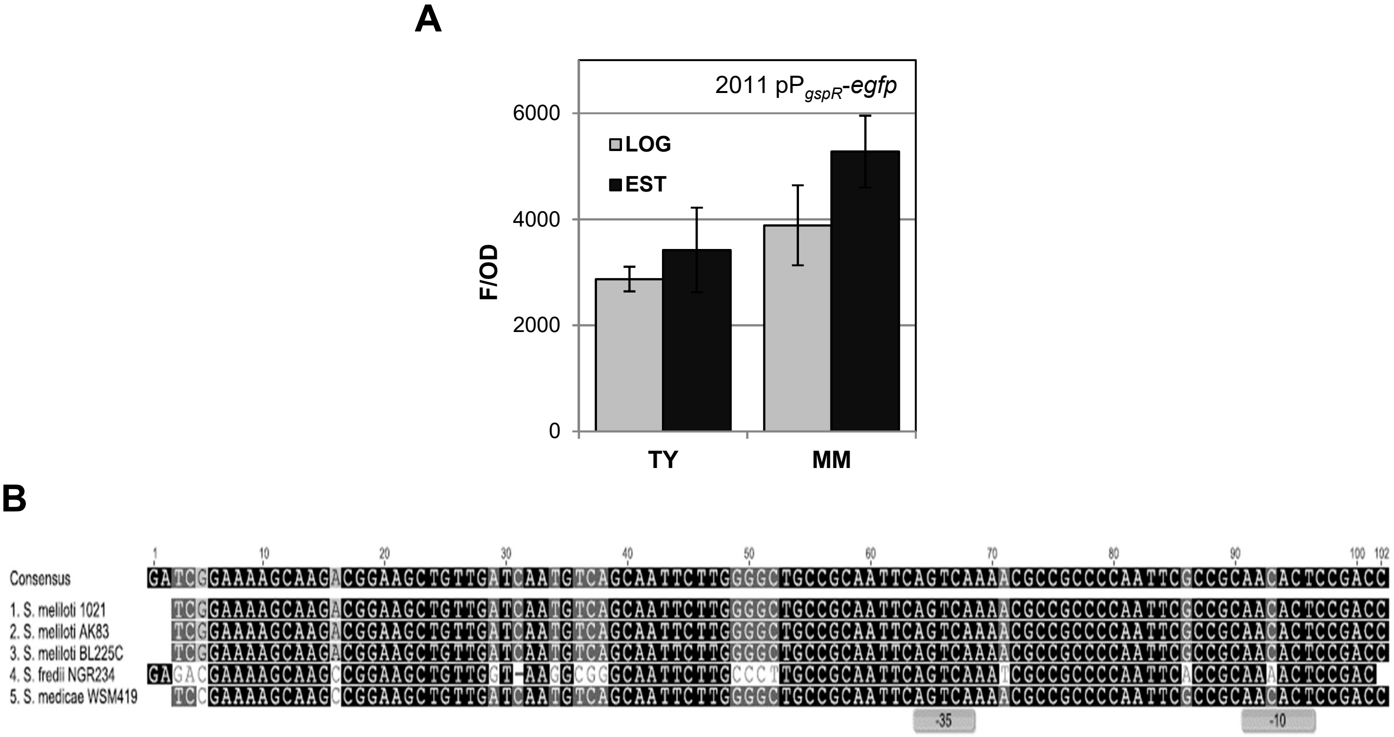

Figure S2. GspR promoter conservation. (A) Fluorescence values of Rm2011 derivative cells harboring pP_{gspR}-egfp in logarithmic and stationary phase of growth. Specific activities were normalized to OD_{600} to yield fluorescence units per unit of optical density (F/OD). Shown are means and standard deviation values of at least three independent measurements of three transconjugants grown in six independent culture. (B) Promoter alignment of the GspR -100 region in different *Sinorhizobium* strains. The -10 and -35 RNA polymerase putative recognition regions are boxed. Nucleotide positions are numbered relative to the TSS. Highly and weakly conserved nucleotides are represented from black to white. Promoter consensus sequences derived from *S. meliloti* are indicated on the top.

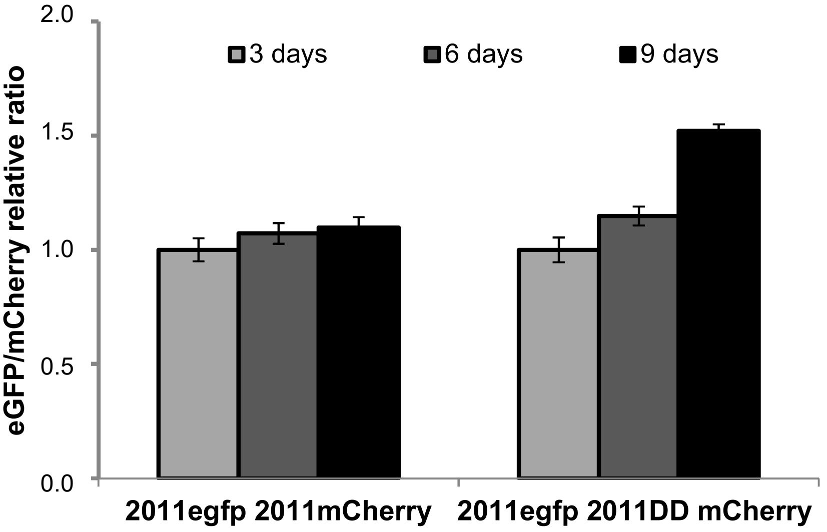

Figure S3. Fitness of Rm2011 wild type vs. 2011DD mutant. Relative eGFP:mCherry fluorescence ratios of 2011egfp mixed 1:1 with either 2011mCherry (control) or 2011DD mCherry cell cultures in minimal medium at the indicated time points. Every 3 days the stationary cultures containing the mixed population were measured and diluted 1000-fold in fresh medium. Standard deviation represents three determinations of four independent cultures.

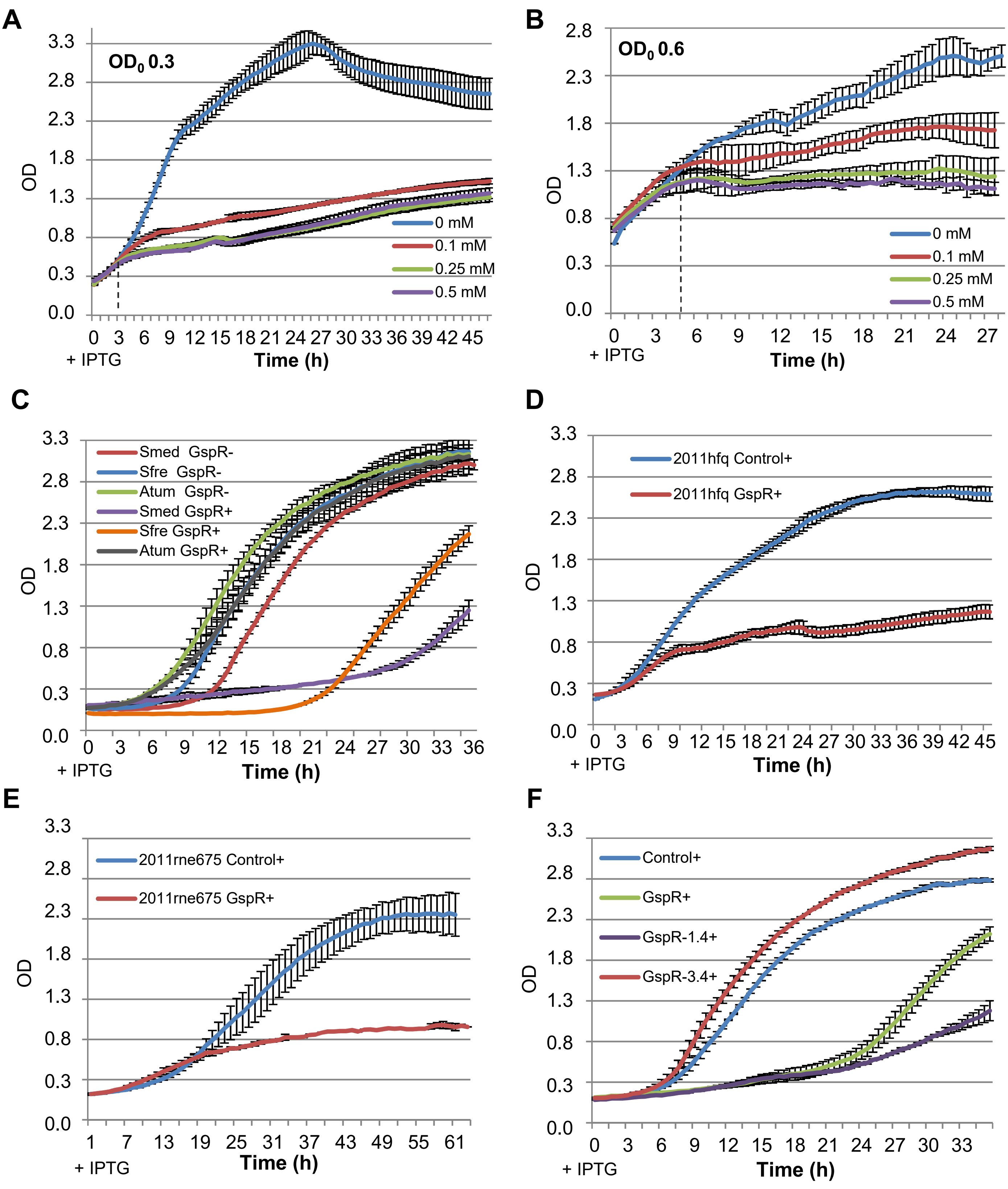

Figure S4. Growth arrest phenotype induced by *gspR* overexpression in different conditions. Growth rates of *Sinorhizobium* and *Agrobacterium* strains cultures in TY-rich medium overexpressing the control RNA gene SmelC812 (Control$^+$), *gspR* (GspR$^+$). Sm2B2019DD cultures after addition of different IPTG concentrations (0 h) to initial OD 0.3 (A) or 0.6 (B). Horizontal dashed line shows the start point of significant growth differences with 0.5 mM IPTG (4 h). *S. medicae* WSM419 (Smed), *S. fredii* NGR234 (Sfred) and *Agrobacterium tumefaciens* C58 (Atum) strains carrying pSKGspR$^+$ without IPTG (GspR-) or after addition of 0.5 mM IPTG (0 h) to OD 0.2 cultures (C). Rm2011hfq (D) and Rm2011rne675 (E) strains after addition of 0.5 mM IPTG (0 h) to OD 0.2 cultures. (F) Sm2B2019DD carrying mutant variants *gspR-1.4* and *gspR-3.4* with 4 nt exchanges in SL1 and SL3, respectively, after addition of 0.5 mM IPTG to OD 0.4.

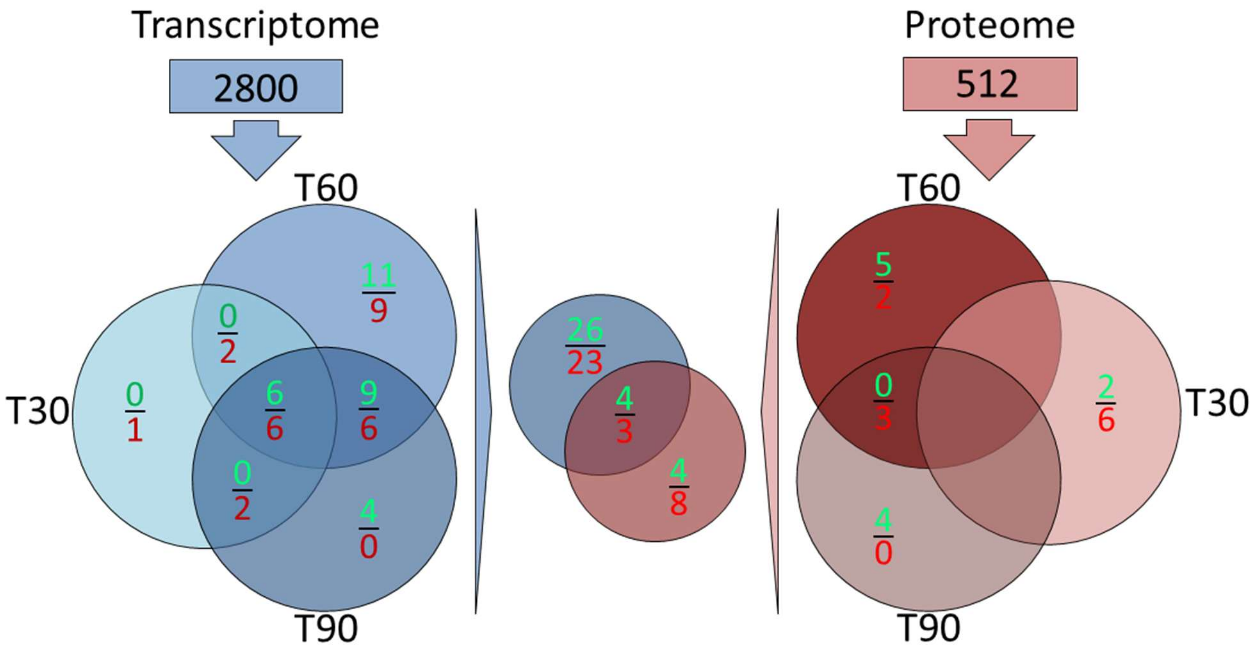

Figure S5. Transcriptome or proteome profiles in the GspR⁺ strain compared to the control strain. Two-circle Venn diagram displays all identified protein-coding genes with significant changes (M-value: ≥1; ≤-1) from both approaches. T30\60\90, samples were prepared for measurement 30, 60, and 90 min after induction of GspR overproduction; green, genes with increased expression pattern; red, genes with decreased expression pattern.

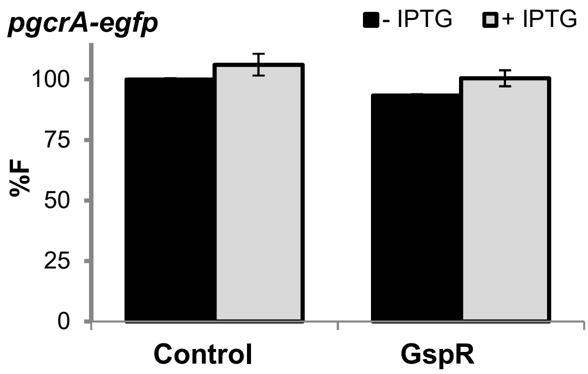

Figure S6. Means of relative fluorescence intensity values of Sm2B2019DD co-transformed with overexpression plasmids carrying control *SmelC812* or *gspR* and the indicated *gcrA* control translational fusion. Fluorescence measurements have been performed as described in Figure 4.

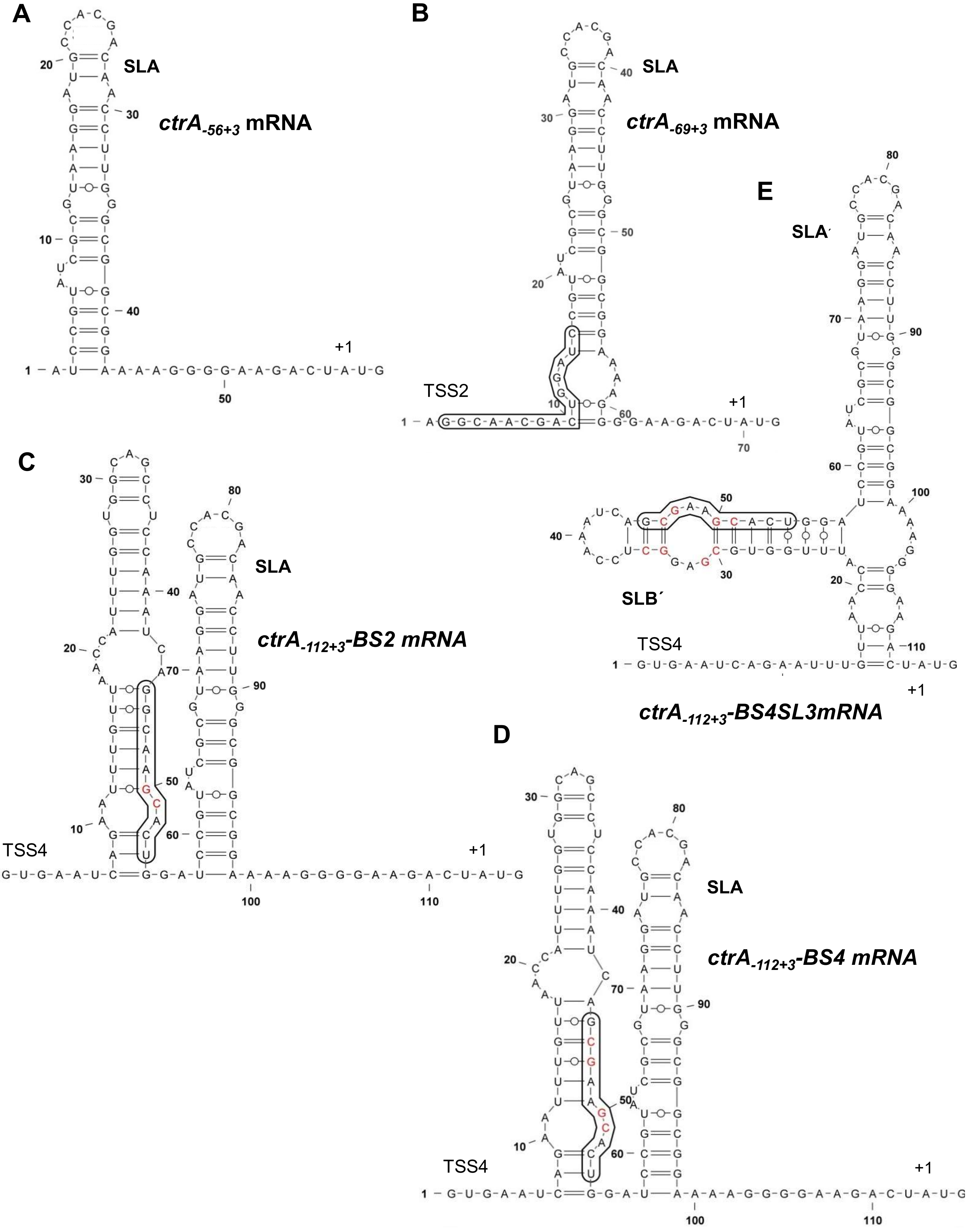

Figure S7. Predicted secondary structures of *ctrA*$_{-69+3}$ (A) *ctrA*$_{-56+3}$ (B) *ctrA*$_{-112+3}$–BS2 (C), *ctrA*$_{-112+3}$–BS4 (D) and *ctrA*$_{-112+3}$ –BS4SLB3 (E) mRNA transcripts exhibiting one two stem loop (SL) structures, denoted SLA and SLB. Nucleotide positions relative to the 5'-end are indicated. The 10-nt GspR interaction region on *ctrA* SLB is boxed. The nucleotide exchanges in the GspR interaction regions on the mRNAs of *ctrA* (*ctrA*-BS.2 and .4) are indicated in red.
